# USP13 drives lung squamous cell carcinoma by switching lung club cell lineage plasticity

**DOI:** 10.1186/s12943-023-01892-x

**Published:** 2023-12-13

**Authors:** Juntae Kwon, Jinmin Zhang, Boram Mok, Samuel Allsup, Chul Kim, Jeffrey Toretsky, Cecil Han

**Affiliations:** 1 Department of Oncology, Georgetown University School of Medicine, Washington D.C, USA; 2 Department of Biochemistry and Molecular & Cellular Biology, Georgetown University School of Medicine, Washington D.C, USA; 3 Division of Hematology and Oncology, Georgetown University School of Medicine, Washington D.C, USA; 4 MedStar Georgetown University Hospital, Washington D.C, USA; 5 Lombardi Comprehensive Cancer Center, Washington D.C, USA; 6 Departments of Pediatrics, Washington D.C, USA

**Keywords:** USP13, c-Myc, Lung squamous cell carcinoma, Lineage plasticity, GEMM

## Abstract

Lung squamous cell carcinoma (LUSC) is associated with high mortality and limited targeted therapies. USP13 is one of the most amplified genes in LUSC, yet its role in lung cancer is largely unknown. Here, we established a novel mouse model of LUSC by overexpressing USP13 on Kras^G12D/+;^ Trp53^flox/flox^ background (KPU). KPU-driven lung squamous tumors faithfully recapitulate key pathohistological, molecular features, and cellular pathways of human LUSC. We found that USP13 altered lineage-determining factors such as NKX2-1 and SOX2 in club cells of the airway and reinforced the fate of club cells to squamous carcinoma development. We showed a strong molecular association between USP13 and c-MYC, leading to the upregulation of squamous programs in murine and human lung cancer cells. Collectively, our data demonstrate that USP13 is a molecular driver of lineage plasticity in club cells and provide mechanistic insight that may have potential implications for the treatment of LUSC.

**Graphical Abstract:**

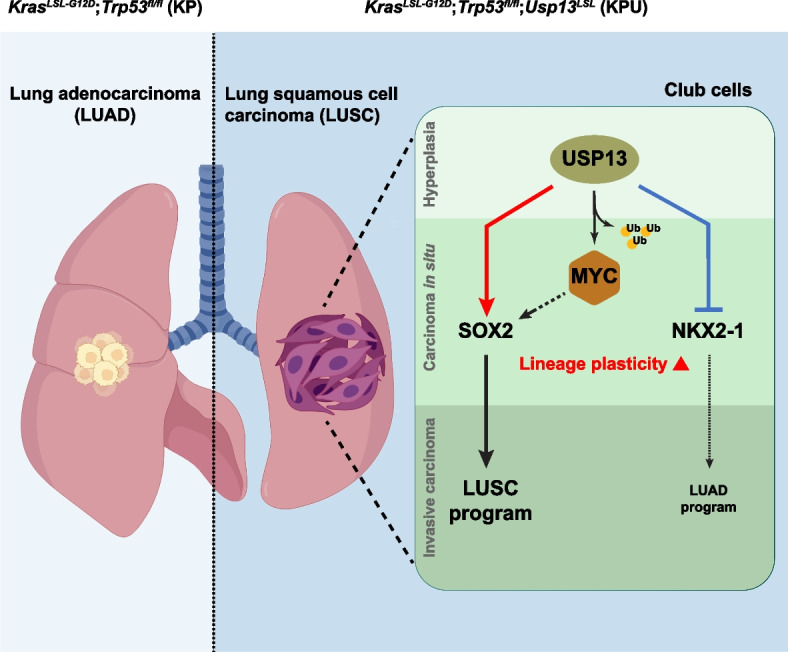

**Supplementary Information:**

The online version contains supplementary material available at 10.1186/s12943-023-01892-x.

## Background

Lung cancer is the most common cancer and is the leading cause of cancer-related death worldwide [1, 2]. Non-small cell lung carcinoma (NSCLC) and small-cell lung carcinoma (SCLC) are the two most frequent lung cancers. NSCLC accounts for more than 85% of lung cancer cases and is classified into lung adenocarcinoma (LUAD, 50%), lung squamous cell carcinoma (LUSC, 30-40%), and large cell carcinomas [1]. Unfortunately, most patients with LUSCs are diagnosed at an advanced stage with high mortality [3]. The lack of targeted therapy specific to LUSC leaves advanced-stage patients with few treatment options. Targeted therapies for LUSC have been challenging due to the high level of tumor heterogeneity, fewer oncogenic mutations identified, a limited mechanistic understanding of oncogenic pathways, and a lack of representative mouse models [3, 4]. Recently, lineage plasticity, the ability of a cell to change from one differentiation state to a different identity, has been linked to intratumoral heterogeneity, histological transition among tumor subtypes, and potential mechanisms of therapeutic resistance in lung cancers [5, 6].

Cell-of-origin influences tumor histotypes, malignancy, lineage plasticity, and tumor microenvironment [7–10]. Multiple stem/progenitor cells in the lung epithelium with distinct capabilities of lineage plasticity have been reported in basal-like stem cells (BSCs) in the trachea, secretoglobin family 1A member 1 (SCGB1A1, also known as CC10)-positive secretory club cells in bronchioles, surfactant protein C (SFTPC, also known as SPC)-positive alveolar type 2 (AT2) pneumocyte cells in the alveolar ducts, and CC10/SPC dual-positive bronchioalveolar stem cells (BASCs) in the bronchioalveolar junctions (BADJs) [11–13]. LUSC is characterized by the expression of genes centered around squamous cell fate decisions and/or the squamous cell differentiation program [3, 14]. The cell of origin for LUSC has been hypothesized to be BSCs, AT2 cells, BASCs, and club cells of small airways [7, 14]. Cell lineage-specifying transcription factors have driven diverse lung cancer types [15]. Specifically, SRY-box transcription factor 2 (SOX2) is a key determinant of squamous cell fate and promotes squamous cell carcinoma of the lung. NK2 homeobox 1 (NKX2-1, or TTF-1) is another key factor for regulating lung lineage transcription programs and is enriched in LUAD [16–18].

Human LUSC develops from normal airway epithelium and progresses through hyperplasia, squamous metaplasia, dysplasia, and carcinoma [19]. However, not all preneoplasia is destined to progress to invasive LUSC, and it is not fully understood which molecular changes establish each stage and drive progression. More than 90% of human LUSC tumors exhibit chromosome 3q26 copy number gains (CNGs) as a genetic hallmark of LUSC [20, 21]. Ch.3q26 CNGs occur in preneoplastic lesions and at a higher frequency in subsequent malignant lesions, suggesting that 3q26 CNGs may be associated with the transition from the premalignant to the invasive LUSC [19, 22]. Notably, this 3q26 amplicon contains *SOX2* and a number of additional potential drivers or modifier genes that may be of biological and therapeutic relevance to LUSC tumorigenesis [20]. While *KRAS* mutations are frequently observed in human LUAD, various studies have also discovered *KRAS* mutations in 1%-6% of LUSC [23–25]. Notably, in up to half of human LUSC tumors, the KRAS driving pathway is activated, most commonly due to transcriptional upregulation and amplification of *KRAS* and the upstream receptor tyrosine kinases *EGFR* (epidermal growth factor receptor) and *FGFR1* (fibroblast growth factor receptor 1) [26]. Nevertheless, it is not known whether *KRAS* mutations contribute to LUSC progression [23].

*USP13* is located on chromosome 3q26 amplicon, alongside the oncogene *PI3CA* (phosphatidylinositol-3-kinase catalytic subunit, α-isoform) and *SOX2* in human LUSC [20]. As a deubiquitinase enzyme, USP13 has been implicated in tumor promotion or suppression by regulating the stability of various substrate proteins via deubiquitination processes [27, 28]. USP13 functions as an oncogenic factor during tumorigenesis in ovarian cancer by regulating cancer metabolism and metastasis [29, 30]. USP13 promotes tumorigenic potential, cell invasion, and cell survival in different human cancers [31–33]. Conversely, USP13 acts as a tumor suppressor by stabilizing PTEN (phosphatase and tensin homolog) in oral squamous cell carcinoma, breast cancer, and bladder cancer [34–37]. These findings highlight that the function of USP13 can be contextually influenced and vary across diverse tumor types. Here, we developed and employed a novel GEMM to elucidate the role of USP13 in lung cancer development. USP13 is sufficient to reprogram lineage plasticity in murine bronchiole club cells and drive invasive squamous cell carcinoma development in the context of oncogenic *Kras* activation and *Trp53* deletion.

## Methods

### Mice

*Usp13*^*LSL/LSL*^ (U) mouse strain was previously described [30]. C57BL/6J *Kras*^*LSL-G12D/+*^ (K) mouse and C57BL/6J *Trp53*^*flox/flox*^ (P) mouse were purchased from The Jackson Laboratory (#008179, #008462). K, KU, KP, and KPU mice were established by breeding with different mice combinations. Genotypes were confirmed with allele-specific primers (Table S1) using tail-ti*p-*derived DNA. Similar numbers of male and female mice with desired genotypes were used as experimental mice, and no histologic differences were observed between male mice and female mice. All mice were housed under standard housing conditions at the Division of Comparative Medicine animal facilities, and all animal procedures were reviewed and approved by the Institutional Animal Care and Use Committee of Georgetown University.

### Cell culture

Isolation of primary lung cancer cells from KP and KPU mice was performed as previously described with minor modifications [38, 39]. At the endpoint of experiments, tumor-bearing mice were sacrificed, and the lungs were perfused by D-PBS. The tissue was briefly rinsed in PBS and transferred to PBS-containing Petri dishes. Tumor lesions were excised with a scissor and minced with a sterile blade. The tumor-containing tissue was incubated with DMEM-supplemented collagenase/dispase (100 ul/ml) (Sigma-Aldrich, Cat#11097113001) and DNase I (40 ul/ml) (Sigma-Aldrich, Cat#11284932001) for 1 h at 37 °C in 5% CO2 cell culture incubator. The reaction was stopped by the addition of a cold medium and filtered by 70 µm cell strainer to generate single-cell suspensions. This mixture was briefly spun in a benchtop centrifuge and the supernatant was discarded. Digested tissue was re-suspended in DMEM supplemented with 10% FBS and 1% penicillin–streptomycin (PS), and washed three times with 1 ml solution prior to plating in a 6-well tissue culture plate. During subsequent culture, fibroblasts were counter-selected by selective trypsinization, and cell clusters with a homogenous morphology were clonally expanded. These clones were further characterized by genotyping PCR and western blotting. Human NSCLC cell lines were obtained from ATCC and cultured in DMEM supplemented with 10% FBS and 1% PS. Except when a different concentration was indicated, the reagents were dissolved in dimethyl sulfoxide (DMSO) and added to the cells at the following concentrations: cycloheximide (CHX; 100 µg/ml) (Thermo Fisher Scientific, Cat#J6690103), MG-132 (10 µg/ ml) (Cayman Chemical, Cat#10012628), Spautin-1 (10 µM) (Selleckchem, Cat#S7888), and 10058-F4 (10 µM) (Selleckchem, Cat#S7153).

### Mouse lung tumor initiation

Anesthetized K, KU, KP, or KPU mice at 7-14 weeks of age were infected by intratracheal intubation [40] with 3x10^7^ pfu of Ad5-CMV-Cre, 5-10 x10^7^ of Ad5-CC10-Cre, or 2.5-5 x10^9^ pfu of Ad5-SPC-Cre adenovirus (University of Iowa). Viruses were administered in a Biosafety Level 2+ room according to Institutional Biosafety Committee guidelines. For immunohistochemistry of murine tissues, KP and KPU mice were sacrificed at 10-14 weeks post-adenoviral infection. K and KU mice were sacrificed at 44-50 weeks post infections to match stages of tumor development and burden. Lung histopathological features and stages were evaluated by a certified thoracic pathologist.

### Immunohistochemistry

For formalin-fixed samples, mouse tissues were fixed in 10% neutral buffered formalin for 24 h at room temperature (RT), washed in D-PBS and transferred to 70% ethanol. Formalin-fixed paraffin-embedded (FFPE) sections at 5 µm were dewaxed, rehydrated and subjected to high-temperature antigen retrieval by boiling 20 min in a 2100 Antigen retriever in 0.01 M citrate buffer (Vector laboratories) at pH6.0. Slides were quenched of endogenous peroxide in 3% H_2_O_2_ for 5 min, then blocked in 2.5% goat serum in TBS/0.08% Tween-20 (TBS-T) for 1 h, and then stained overnight with primary antibias in blocking buffer (2.5% goat serum). M.O.M Elite® Immunodetection Kit (Vector Laboratories, Cat# PK-2200) was used for primary mouse antibodies following the manufactural instruction. HR*P-*conjugated secondary antibody (Vector Laboratories) was used at 1:200 dilution in TBS-T, incubated for 30 min at RT followed by DAB staining (Vector Laboratories, Cat#SK-4100). The primary antibodies include: USP13 (Santa Cruz, Cat# sc-514416) 1:600, NKX2-1 (Abcam, Cat# ab76013) 1:2000, SPC (Abcam, Cat# ab211326) 1:500, SOX2 (CST, Cat# 3728) 1:400, Cytokeratin 5 (Thermo Fisher Scientific, Cat# MA5-17057) 1:2000, MYC (Abcam, Cat# ab32072) 1:400. Images of H&E and IHC-stained slides were acquired on the inverted microscope with microscope cameras DFC7000 T (Leica Microsystems). Human tissue microarray (TMA) sections were purchased from Biomax (Cat#HLugA150CS02, LUAD; Cat#HLug-Squ150Sur-02, LUSC; Cat#LC2162, NSCLC). For IHC score quantification, images were digitally scanned with Aperio GT 450. IHC score was quantified by Fiji software. IHC score = % of positive cells multiplied by intensity. IHC-stained slides were digitally scanned with Aperio GT 450 to quantify human tissue microarray. Tumor regions were manually annotated, and image analysis algorithms were applied to tumor regions. The algorithms distinguish cells as positive or negative based on the staining intensity per cell.

### Immunofluorescence

Immunofluorescence staining of mouse lung tumors was conducted using primary antibodies of NKX2-1 (Abcam cat# ab76013), SOX2 (Santa Cruz, Cat# sc-365823), CC10 (Santa Cruz, Cat# sc-365992), and SPC (Abcam, Cat# ab211326) flowed by Alexa Fluor™ Plus 594 and Alexa Fluor™ Plus 488-labeled secondary antibodies (Thermo Fisher Scientific, Cat# A32742 and Cat# A32731). Slides were then mounted with DAPI-containing Fluoroshield Mounting Medium (Sigma-Aldrich, Cat#ab104139), visualized under the microscope (Leica Microsystems, Cat#DFC7000 T)

### Immunoblotting

Cell pellets were directly used or flash frozen and stored at -80℃ until use. Total protein lysates were prepared with 1% N*P-*40 lysis buffer, separated via SDS-PAGE, and transferred to a PVDF membrane. Membranes were blocked for 1 h in 5% milk followed by overnight incubation with primary antibodies directed against USP13 (Santa Cruz, Cat# sc-514416), NKX2-1 (Abcam, Cat#ab76013), SOX2 (CST, cat#14962), MYC (Abcam, Cat#ab32072), CK5 (CST, Cat#71536), p63 (Abcam, Cat#ab124762), SPC (Abcam, Cat#ab211326), β-actin (Santa Cruz, Cat# sc-47778), FLAG-tag (Sigma-Aldrich, Cat#F1804), Myc-tag (CST, Cat#2276), HA-tag (Thermo Fisher Scientific, Cat#14-6756-63) at 4℃. Membranes were washed for 3 x 10 min at RT in TBS-T. Mouse and rabbit HR*P-*conjugated secondary antibodies (Jackson ImmunoResearch, 1:10,000) were incubated for 50 min in 1% milk at RT followed by washing 3 x 10 min at RT in TBS-T. Membranes were exposed to Clarity Western ECL Substrate (Bio-rad, Cat# 1705061) and detected on ChemiDoc MP Imaging System (Bio-Rad, Cat#17001402). Quantification of immunoblots was performed using ImageJ.

### Quantitative real-time PCR

Total RNA was extracted from cells using RNeasy Plus Micro Kit (Qiagen), processed for cDNA synthesis using the Reverse Transcription Kit (Applied Biosystems, Cat#4374966), and subjected to the quantitative RT-PCR using SYBR Green Mix (Applied Biosystems, Cat#A25742). The expression genes were normalized to the expression of human actin as a housekeeping gene. Primers for qPCR: human USP13 (5’-ACAGCCAGGAGAGGAAGAAC-3’ and 5’-TCAATTGGTTC ATCAGGCGA-3’), human MYC (5’- CCTGGTGCTCCATGAGGAGAC-3’ and 5’- CAGACTCTGACCTTTT GCCAGG-3’), and human β-actin (5’-CACCATTGGCAATGAGCGGTTC-3’ and 5’-AGGTCTTTGCGGA TGTCCACGT-3’).

### Knockdown and overexpression of USP13 and MYC

Cells were seeded into a 6-well plate at a 60% confluency for transient overexpression and transfected with desired plasmids using Lipofectamine 3000 (Thermo Fisher Scientific, Cat#L3000001). The transfection was performed according to the manufacturer’s recommended protocol, using a 3:1 ratio of Lipofectamine/DNA. The next day after transfection, the medium was changed.

To generate shRNA constructs against USP13, the following sequences are targeted: shUSP13-1: 5’- AAGGGAACATGTTGAAAGACAT-3’ and shUSP13-2: 5’-GCATGTCGCAAGGCTGTGT-3’. Those sequences were cloned into pLKO.1-puro (Addgen, Cat#8453) plasmid. pLKO.1-puro USP13 shRNA plasmids were confirmed by direct sequencing. pCDH-puro-cMYC was purchased from Addgene (Cat# 46970), and pCDH-puro-USP13 was previously generated [29]. For the generation of the high-titer virus, HEK293T cells were transfected with a three-plasmid system including psPAX2 (Addgene, Cat# 12260), pMD2.G (Addgene, Cat#12259), and lentiviral plasmid. Viruses were harvested at 48 and 72 h post-transfection and stored at -80℃ until use. To establish stable cell lines, lentiviral transduced cells were selected with puromycin (2–3 µgml^–1^) 48 h post-infection, and individual colonies were propagated and validated by western blotting (protein).

To knock down MYC protein, the following control siRNA (Cat#sc-37007) and siMYC RNA (Cat#sc-29226) were purchased from Santa Cruz and transfected into cells using siRNA Transfection Reagent (Cat#sc-29528) followed by the manufacturer’s instructions.

### Mouse tumor bulk RNA-seq

Anesthetized KP and KPU mice at 7-14 weeks of age were infected by intratracheal intubation with 3x10^7^ pfu of Ad5-CMV-Cre adenovirus. After 12 weeks, mice were sacrificed, and lungs were perfused with 20 mL of PBS. Large tumors were dissected and homogenized and RNA was extracted using RNeasy Plus Micro Kit according to manufacturer’s instructions (Qiagen, Cat#74134). Sequence reads were trimmed to remove possible adapter sequences and nucleotides with poor quality using Trimmomatic v.0.36. The trimmed reads were mapped to the Mus musculus GRCm38 reference genome available on ENSEMBL using the STAR aligner v.2.5.2b. Reads were quantified using HTSeqv0.6.1. Rank-log transformed normalized counts from DESeq2 were used as inputs for PCA, GSEA, and IPA analysis [41].

### Pathway enrichment

Gene set enrichment analysis (GSEA) was performed using GSEA 4.0 (Broad) with gene-set permutation [42]. Gene set annotations were taken from Molecular Signatures Database (MSigDB v7.0.1) [43]. The significance level of enrichment was evaluated using permutation test, and the *p-*value was adjusted by Benjamini–Hochberg procedure. Any enriched gene sets with adjusted *p-*value ≤ 0.05 were regarded as significant. Pathway and upstream factor analysis were performed through Ingenuity Pathway Analysis (IPA) software (Qiagen) [44]. Genes were analyzed using Core Analysis to identify statistically significant canonical pathways via right-tailed Fisher’s exact test. IPA pathway databases also estimate regulatory direction for a subset of the canonical pathways and statistical significance with activation z-score. Additionally, using the Comparison Analysis tool, the respective datasets (with padj < 0.01) were compared and a heatmap illustrating the activation z scores for each upstream factor in each dataset.

### Immunoprecipitation

HEK293T cells were transiently transfected using Lipofectamine 3000. Cell lysis was carried out with lysis buffer (50 mM Tris pH 8.0, 150 mM NaCl, 1 mM EDTA, and 0.1% N*P-*40) supplemented with protease and phosphatase inhibitors. Immunoprecipitation was performed using Protein G agarose beads (Roche, Cat#11719416001), 1 µg of the specific Ab, and 500 µg of protein lysates. Beads were washed three times with immunoprecipitation buffer, boiled for 10 min in reducing 4X SDS Laemmli Sample Buffer (Bio-Rad, Cat#1610747), and denatured at 95 ℃ for 10 min. Total cell lysates and immunoprecipitants were separated by SDS–polyacrylamide gel electrophoresis and analyzed by western blotting

### Ubiquitin assay

HEK293T cells were transiently co-transfected with indicated plasmids. After 48 h, cells were treated with 10 µg/ml MG-132 (Cayman Chemical, Cat#10012628) for 6 h before being collected. Cells were lysed and incubated with ANTI-FLAG® M2 Affinity Gel (Sigma-Aldrich, Cat#A2220). Beads were washed three times with immunoprecipitation buffer, boiled for 10 min in reducing 4X SDS Laemmli Sample Buffer and denatured at 95 ℃ for 10 min. Total cell lysates and immunoprecipitants were separated by SDS–polyacrylamide gel electrophoresis and analyzed by western blotting.

### Quantification and statistical analysis

Statistical analyses were conducted using GraphPad Prism 8 (GraphPad Software, CA, USA). To compare two groups, a two-tailed Student's t-test was used with significance considered at *P <* 0.05. Correlation between USP13 and MYC at mRNA and protein level was measured by Pearson correlation. Data were presented as means with SEM unless otherwise specified. Survival data was obtained from the Kaplan-Meier plotter (https://kmplot.com/). All histopathological results were blinded to pathologists, and the evaluation reports, including the classification of different groups, are provided by pathologists.

## Results

### USP13 is highly amplified in NSCLC and correlated with poor prognosis

**Fig. 1:**
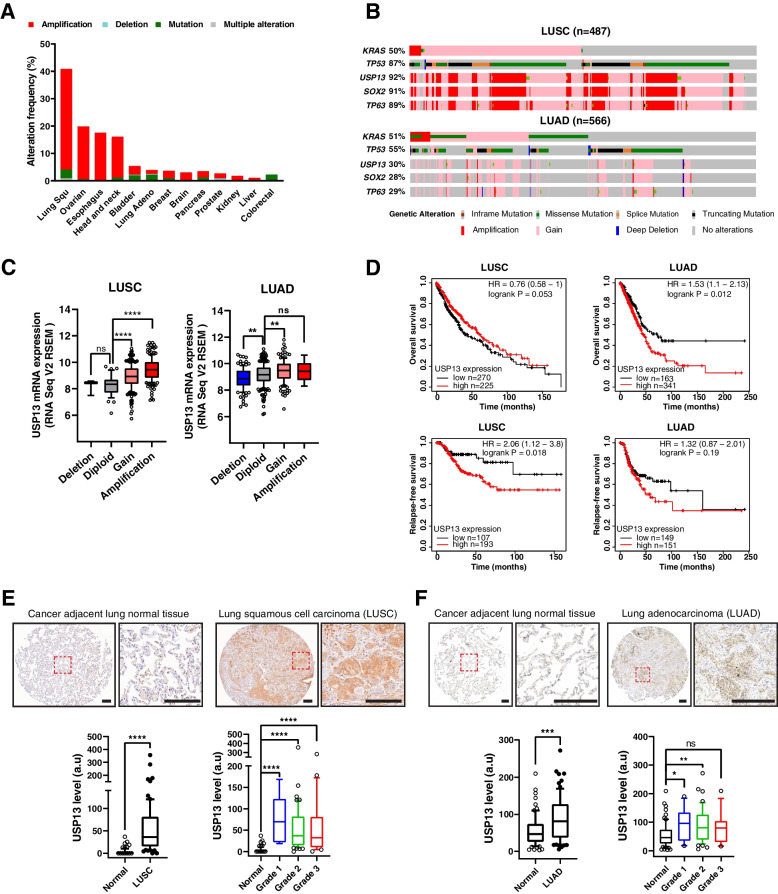
*USP13* is highly amplified in NSCLC and associated with poor survival. **A** Genomic alteration frequency of *USP13* gene in multiple cancer types. **B** Oncoprint mutation profile of *KRAS*, *TP53*, *USP13*, *SOX2,* and *TP63* in LUSC (top) and LUAD (bottom). *USP13*, *SOX2,* and *TP63* genes are co-amplified in NSCLC. **C** The correlation between mRNA expression and copy number variations (CNVs) of *USP13* in LUSC (left) and LUAD (right). **D** Kaplan-Meier plots of NSCLC patients stratified by *USP13* expression: Overall survival (top) and relapse-free survival (bottom) in LUSC (left) and LUAD (right). *p-*values were calculated using the log-rank test. **E** USP13 protein level in human LUSC. Representative IHC-staining images in a tissue microarray of adjacent normal tissue and LUSC (top) (scale bar, 200 µm). The red boxed areas on the left images were magnified and shown on the right. Relative USP13 protein levels in LUSC (*n=*75) and normal tissues (*n=*75) (bottom, left). USP13 expression levels in different LUSC grades (bottom, right). Grade 1 (*n=*5), grade 2 (*n=*50) and grade 3 (*n=*20). **F** USP13 protein level in human LUAD. Representative IHC-staining images in a tissue microarray of adjacent normal tissue and LUAD (top) (scale bar, 200 µm). The red boxed areas on the left images were magnified and shown on the right. Relative USP13 protein levels in LUAD (*n=*75) and normal tissues (*n=*75) (bottom left). USP13 expression levels in different LUAD grades (bottom, right). Grade 1 (*n=*11), grade 2 (*n=*45) and grade 3 (*n=*19). a.u., arbitrary unit. In (**C**), (**E**), and (**F**), boxes indicate the 10-90 percentile. Two-tailed unpaired t-tests, ns = not significant, *p < 0.05, **p < 0.01, ***p < 0.001, ****p < 0.0001

The *USP13* gene copy number is highly amplified in human LUSC and several other cancers, including ovarian cancer, esophageal cancer, and head and neck cancer (Fig. 1A). Among 50 human ubiquitin-specific peptidases (USPs), *USP13* was specifically amplified in LUSC patients (Fig. S1A). The Cancer Genome Atlas (TCGA) genomics revealed *USP13* amplification (≥ 5 copies) or gain (1-3 copies) in 91% (427 cases) of LUSC and 29% (145 cases) of LUAD (Fig. 1B). *USP13* is closely located with squamous lineage factors SOX2 and *TP63* within chromosome 3q26-28 locus and coamplified in LUSC and LUAD (Fig. 1B, Fig. S1B). Over half of the *USP13* copy number gain/amplification co-occurred with *KRAS* gene alteration in LUSC and LUAD (Fig. 1B). Interestingly, the mRNA expression of LUSC markers (*KRT16*, *KRT17*, *UPK1B*, and *ALOXE3*) was upregulated in *USP13*-amplified LUAD with *KRAS* mutations (Fig. S1C). The *USP13* mRNA level elevated with increasing gene copy number in both LUSC and LUAD (Fig. 1C). LUSC patients with high *USP13* mRNA expression tended to have poor relapse-free survival (*p <* 0.05). LUAD patients with high *USP13* expression showed reduced overall survival (*p <* 0.05) (Fig. 1D). Immunohistochemistry (IHC) analysis in human LUSC and LUAD tissue microarrays revealed that while USP13 protein is barely expressed in normal human lung tissues, LUSC samples have ∼16.7-fold higher expression levels of USP13 (*p <* 0.0001). USP13 protein was increased in all grades of LUSC patients (Fig. 1E). LUAD tissue samples showed an ~1.5-fold higher expression level of USP13 than normal adjacent tissues (*p <* 0.001) (Fig. 1F).

### USP13 drives squamous cell carcinoma development in* Kras*/*Trp53*-mutant mouse lung

**Fig. 2:**
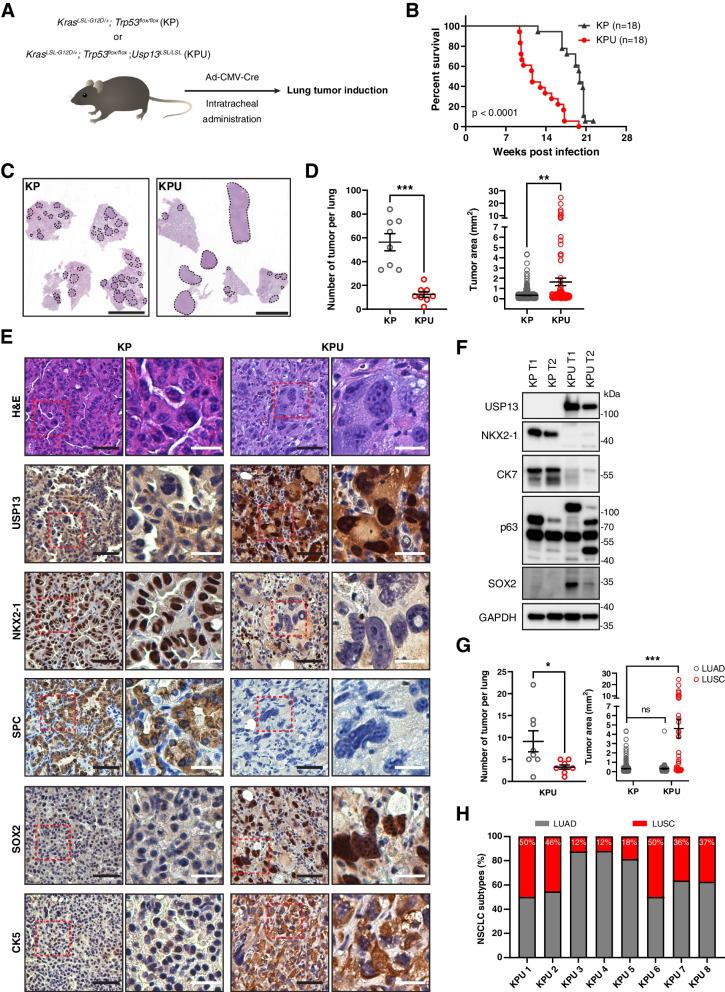
USP13 overexpression drives LUSC development in the KP mouse model. **A** Schematic of the experimental procedure using KP and KPU mouse model. KP or KPU mice were infected with the Adeno-CMV-Cre virus (Ad5-CMV-Cre) via intratracheal administration. **B** Survival analysis of KP and KPU mice upon virus infection. The *p-*value was calculated using a log-rank test. **C** Representative hematoxylin and eosin (H&E) staining of KP and KPU lung at 12 weeks post-viral infection. Dotted lines indicate tumor nodules. Scale bar = 5 mm. **D** Quantification of individual tumor number and area in KP and KPU mice at 10-14 weeks post-Ad5-CMV-Cre virus infection (*n=*8 mice/group). **E** Representative images for H&E, USP13, NKX2-1, SPC, SOX2, and CK5 IHC stains of the KP and KPU tumors. The red boxed areas on the left images were magnified and shown on the right. Black scale bar = 50 µm, White scale bar = 20 µm. (F) Western blot showing the expression of USP13, NKX2-1, CK7, p63, and SOX2 in KP and KPU tumors. GAPDH is a loading control. **G** Quantification of tumor number and area for LUAD and LUSC components in KPU mice. *n=*8 for each group. **H** The ratio of NSCLC subtypes in each KPU mouse (*n=*8). In (**D**) and (**G**), error bars indicate mean ± SEM. Two-tailed unpaired t-tests, *p < 0.05, **p < 0.01, ***p < 0.001

To investigate the role of USP13 in the 3q26 amplicon in lung tumorigenesis, we crossed a conditional *Usp13* knock-in (KI) mouse model (named *Usp13*^*LSL/LSL*^, U) with *Kras*^*G12D/+*^ (K) and *Trp53*^*flox/flox*^ (P) mice to generate the *Kras*^*G12D/+*^; *Trp53*^*flox/flox*^; *Usp13*^*LSL/LSL*^ (KPU) model (Fig. S2A). Lung tumor development was induced via intratracheal administration of adenovirus expressing Cre recombinase (Ad5-CMV-Cre), leading to activation of oncogenic *Kras*, homozygous deletion of *Trp53*, and overexpression of *Usp13* in murine lung epithelium (Fig. 2A). First, we observed that the survival of KPU mice was noticeably shorter than that of *Kras*^*G12D/+*^; *Trp53*^*flox/flox*^ (KP) mice (Fig. 2B). Tumors were detectable in all lung lobes at 10-14 weeks post Ad5-CMV-Cre viral induction in both KP and KPU mice. Despite developing fewer tumor nodules, the sizes of each tumor driven by KPU were significantly larger than those driven by KP (Fig. 2C and D). Conditional activation of oncogenic *Kras*^*G12D*^ and homozygous *Trp53* targeting in the mouse (KP) lung is a well-established mouse model of human LUAD [45]. Indeed, LUAD-related histological features were observed in KP tumors (Fig. S2B). Surprisingly, KPU tumors driven by *Usp13* overexpression exhibited squamous characteristics in their histology, such as keratin material and intercellular bridges (Fig. S2C). Furthermore, KPU tumors displayed heterogeneous histological features, such as SCLC- or sarcoma-like characteristics, in a few lesions (Fig. S2C).

We further examined KP and KPU tumors using IHC analyses. KP tumors expressed LUAD markers, including NKX2-1 and SPC (Fig. 2E). KPU tumors exhibited strong expression of squamous markers (SOX2 and CK5), while adenocarcinoma markers were dramatically downregulated (Fig. 2E). We also confirmed the differential expression of LUAD and LUSC markers in KP and KPU tumor lysates by immunoblot analysis. NKX2-1 and CK7 signals were downregulated, while altered expression of the p63 isoform and upregulated SOX2 were detected in KPU tumor lysates (Fig. 2F). Based on IHC characteristics, we classified the tumor subtype in each tumor nodule of KP and KPU lungs. All KP tumors were determined to be adenocarcinoma (Fig. 2G). Two major lung subtypes, LUAD and LUSC, were observed in 100% of KPU mice with predominantly squamous histology in the large size of tumor nodules (Fig 2G and H). The small size of KPU tumors exhibited components of LUAD similar to KP tumors (Fig. S2D). Next, we wondered whether overexpression of USP13 induces LUSC development in the *Kras*^*G12D/+*^ background (K). We compared lung tumors in K mice [46] and *Kras*^*G12D/+*^; *Usp13*^*LSL/LSL*^ (KU) mice after Ad5-CMV-Cre viral infection (Fig. S2E). Similar to KPU mice, KU mice developed fewer tumor nodules with increased tumor size than K mice (Fig. S2F). However, none of the LUSC components was observed in tumors of KU mice (Fig. S2G), suggesting that depletion of TP53 is critical for LUSC development driven by USP13. Collectively, these data revealed that overexpression of USP13 drives LUSC development in the context of oncogenic *Kras* activation and deletion of *Trp53*.

### Lineage reprogramming pathways are enriched in KPU tumors

**Fig. 3:**
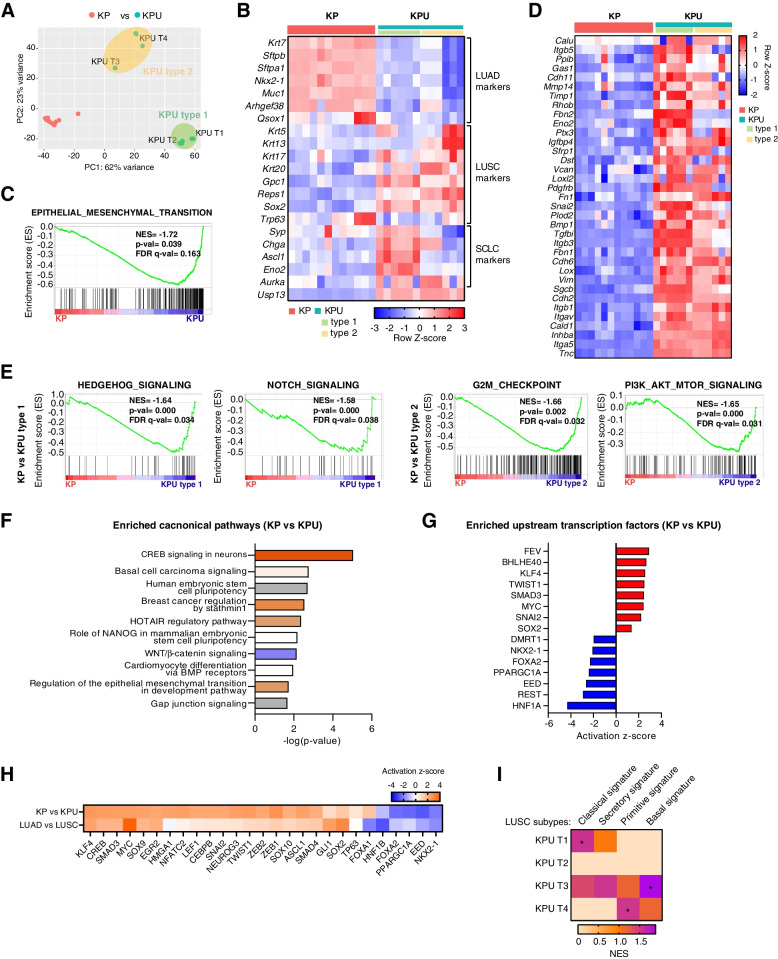
Transcriptomic characterization of KPU tumors. **A** Principal component analysis on the transcriptome of KP and KPU samples. **B** Heatmap showing the expression of marker genes for LUAD, LUSC, and SCLC in KP and KPU tumors. **C** GSEA plot of the curated epithelial-to-mesenchymal transition (EMT) hallmark gene set from MSigDB using RNA-seq data of tumors from KP and KPU mice. **D** Heatmap showing genes significantly upregulated in the “EMT” gene set from (**C**). **E** Enrichment plots of hallmark gene set from MSigDB collection in KP versus KPU type 1 tumors (left) and KP versus KPU type 2 tumors (right) by GSEA analysis. **F** IPA analysis of RNA-seq data showing the top ten canonical pathways enriched in KPU tumors compared with KP tumors. Each bar's color indicates predicted pathway activation or inhibition; Orange, positive z-score (activation); white, zero z-score; blue, negative z-score (inhibition); gray, no pattern. **G** Upstream regulator analysis exhibiting the predicted activation status of transcription factors in KPU tumors. A positive z-score indicates activation and a negative z-score indicates inactivation. **H** Heatmap visualization of IPA upstream regulators altered in KP versus KPU tumors and LUAD versus LUSC patients. The z-score predicts activation (orange) and suppression (blue). **I** Heatmap to show the LUSC subtypes of KPU tumors by the signature gene sets. Row for samples, columns for LUSC subtypes. Asterisk indicates *p <* 0.05

To analyze the molecular characteristics of KPU tumors, we conducted bulk RNA sequencing. Compared to KP tumors, the KPU tumor transcriptome exhibited greater heterogeneity and was classified into two distinct clusters (KPU type 1 and type 2) (Fig. 3A). Consistent with histopathological analysis, the expression levels of LUSC marker genes were increased, while the levels of LUAD markers were downregulated in KPU tumors (Fig. 3B), which resembles the molecular signature of human LUSC (Fig. S3A). Notably, KPU type 1 tumors exhibited upregulation of SCLC and LUSC marker gene expression together (Fig. 3B). We identified 1,050 differentially expressed genes (DEGs) in KPU tumors compared to KP tumors (657 upregulated and 393 downregulated; |log2FC| ≥ 3.5, *p <* 0.01, FDR < 0.01) (Fig. S3B; Table S2). Gene set enrichment analysis (GSEA) with hallmark gene sets revealed that KPU tumors were highly enriched in the epithelial-mesenchymal transition (EMT) pathway (Fig. 3C; Table S3). Many EMT-related genes, such as *Calu*, *Fn1*, *Vim*, and *Snai2*, were upregulated in KPU tumors (Fig. 3D). Enhanced gene sets for KP tumors include xenobiotic metabolism, KRAS signaling down, and peroxisome (Fig. S3C).

We further found that different signaling pathways were enriched in KPU type 1 and type 2. The Hedgehog and Notch signaling pathways were enriched in KPU type 1, and G2/M checkpoint and PI3K/AKT/mTOR signaling were enriched in KPU type 2 (Fig. 3E). The human LUSC transcriptome showed gene set enrichments related to E2F and MYC targets, along with G2/M checkpoint gene sets, compared to human LUAD (Figure S3D). Ingenuity pathway analysis (IPA) of KP and KPU transcriptomes indicated that USP13 overexpression led to alteration of gene expression related to CREB signaling, basal cell carcinoma signaling, stem cell pluripotency, and EMT (Fig. 3F; Table S4). These pathways were also commonly enriched in human LUSC data compared to LUAD data (Fig. S3E).

In IPA upstream regulator analysis, KPU tumors were predicted to activate gene networks regulated by TWIST1, SNAI2, SOX2, and MYC transcription factors that drive EMT, squamous, and lineage reprogramming (Fig. 3G) [47, 48]. On the other hand, LUAD lineage-specific transcription factors (NKX2-1 and FOXA2) [49] were expected to be inactive in KPU tumors (Fig. 3G). A comparison analysis of KPU tumors and human LUSC found that they share common transcription factors that are either coactivated or inactivated (Fig. 3G and H, Fig. S3F). Human LUSCs have been classified into four subtypes by distinct transcriptomic features: primitive, classical, secretory, and basal [50]. KPU T1, KPU T3, and KPU T4 exhibited signatures similar to those of classical, basal, and primitive human LUSCs, respectively (Fig. 3I). In summary, KPU tumors exhibit similar molecular characteristics to human LUSC and display strong tumor plasticity and lineage reprogramming activity, which seems to be associated with the upregulation of squamous lineage markers (*SOX2*), downregulation of *NKX2-1 and FOXA2*, and activation of pluripotency factors such as *MYC, NANOG, and KLF4*.

### USP13 and MYC exhibit a positive correlation in human LUSC and KPU squamous tumors

**Fig. 4:**
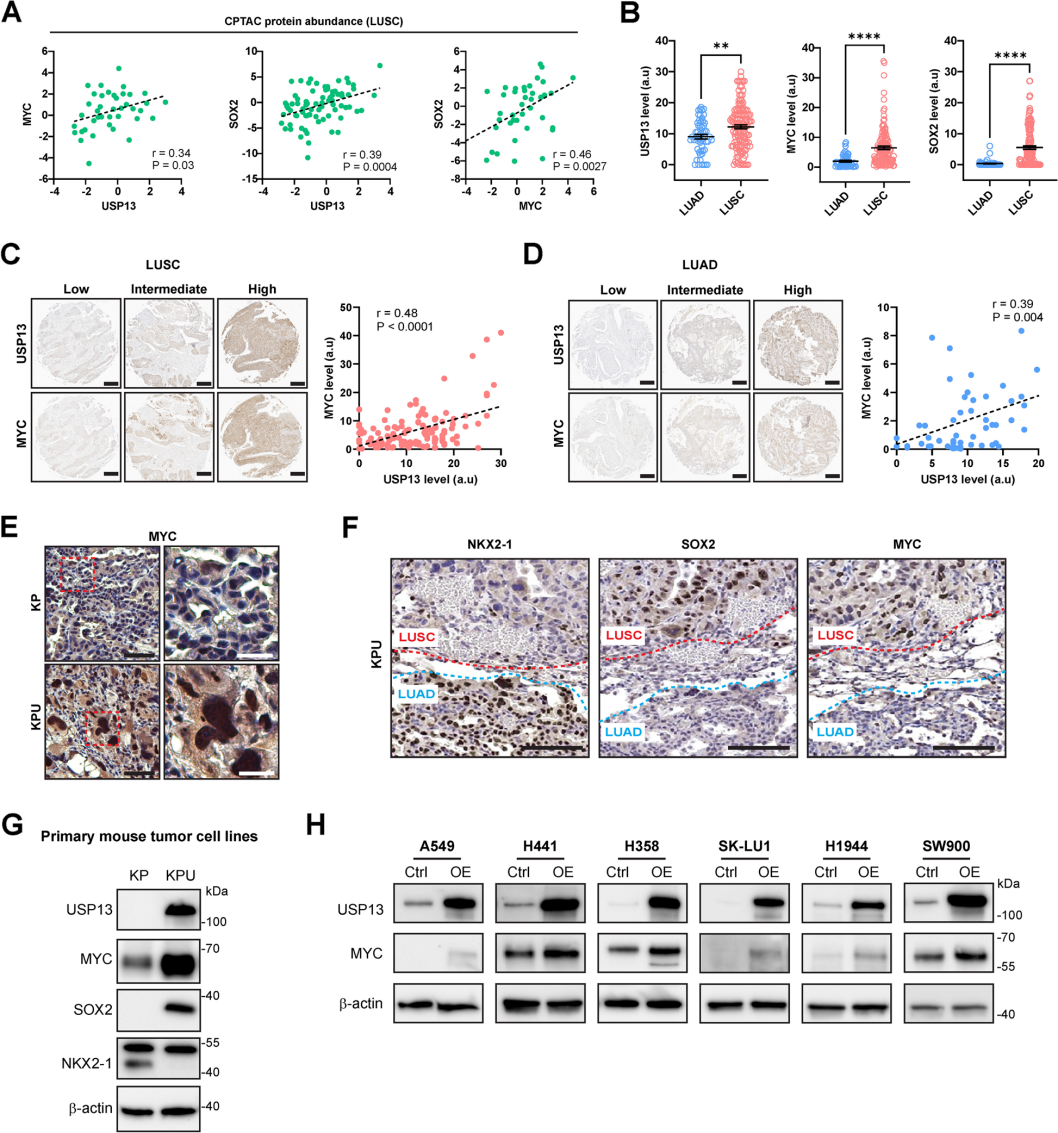
MYC protein is upregulated in lung cancer by USP13. **A** Correlation between USP13 and MYC expression (left), USP13 and SOX2 expression (center), and MYC and SOX2 expression (right) in LUSC. Data was obtained from cBioportal (CPTAC, Cell 2021) (*n=*80). Axis represents the protein abundance ratio. **B** IHC score of USP13, MYC, and SOX2 in LUAD and LUSC patients. Data are shown as means ± SEM. **p < 0.01, and ****p < 0.0001 (unpaired two-tailed t-test). **C** IHC analysis of USP13 and MYC protein abundance in LUSC samples (left) (*n =* 130) and their correlation (right). **D** IHC analysis of USP13 and MYC protein abundance in LUAD samples (left) (*n =* 51) and their correlation (right). **E** Representative images for IHC staining of MYC in the KP and KPU tumors post-Ad5-CMV-Cre infection. The red boxed areas on the left images were magnified and shown on the right. Black scale bar = 50 µm, white scale bar = 20 µm. **F** Expression of NKX2-1, SOX2, and MYC in LUAD and LUSC components in Ad5-CMV-Cre infected KPU lungs. Scale bar = 200 µm. See also Figure S4H. **G** Western blot of endogenous USP13, MYC, SOX2, NKX2-1, and β-actin in KP and KPU mouse cell lines. **H** Western blot of USP13, MYC, and β-actin in human NSCLC cells with or without USP13 overexpression. In (**A**), (**C**), and (**D**), data are analyzed using the Pearson correlation coefficient

Our transcriptomic analysis suggests enhanced MYC activation and its mRNA expression in KPU tumors and human LUSCs (Fig. 3H; Table S2); therefore, we investigated the relationship between USP13 and MYC in human NSCLC. There were positive correlations among USP13, MYC, and SOX2 at the protein level in human LUSC Clinical Proteomic Tumor Analysis Consortium (CPTAC) proteomic data (Fig. 4A). MYC and SOX2 protein abundance data were not available in the LUAD CPTAC dataset. The *USP13* mRNA level was also positively correlated with *MYC* or *SOX2* expression in human LUSC but not in LUAD (Fig. S4A and B). We further examined USP13, MYC, and SOX2 protein expression in human NSCLC tissue microarrays using IHC. USP13, MYC, and SOX2 were significantly higher in LUSC tissues than in LUAD tissues (Fig. 4B, Fig. S4C-E). A positive correlation between MYC and USP13 protein expression levels was found in both LUSC and LUAD tissues (Fig. 4C and D, Fig. S4F). In addition, there were positive correlations between USP13 and SOX2, as well as MYC and SOX2, in LUSC tissues (Fig. S4G).

We next investigated the expression of MYC in KP and KPU tumors. MYC was strongly expressed in KPU tumors, predominantly in the nucleus (Fig. 4E). In particular, MYC was only elevated in the LUSC components but not in the LUAD components of KPU tumors (Fig. 4F and Fig. S4H), suggesting that elevated MYC expression might be implicated in the progression of LUSC in KPU lungs. The established primary KPU lung cancer cells expressed higher levels of MYC than KP primary cells (Fig. 4G). Notably, exogenous USP13 overexpression induced the upregulation of MYC expression levels in multiple human NSCLC cell lines (Fig. 4H). These data reveal that USP13 is associated with the level and/or activity of MYC in lung cancer and imply the potential contribution of USP13 and MYC to LUSC development.

### USP13 stabilizes MYC protein via its deubiquitinating enzyme activity

**Fig. 5:**
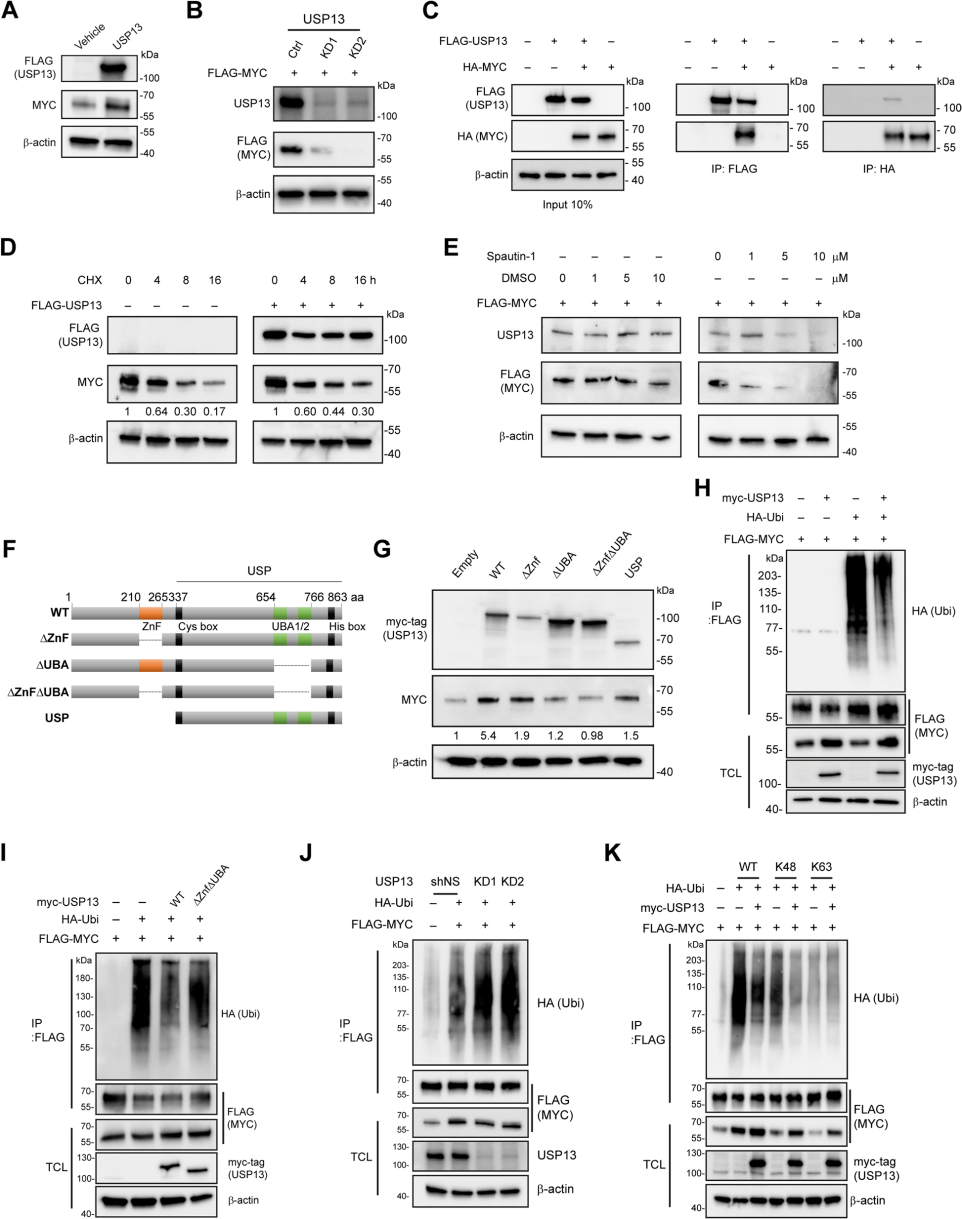
USP13 stabilizes MYC protein via its deubiquitinating enzyme activity. **A** Immunoblot analyses of HEK293T cells with or without USP13 overexpression were performed with the indicated antibodies **B** Western blot showing USP13 and FLAG (MYC) and β-actin in control and USP13 knockdown cells following transfection with an empty vector or FLAG-MYC. **C** Co-immunoprecipitation (Co-IP) of exogenous FLAG-USP13 and HA-MYC in HEK293T cells. **D** CHX chase assay (100 µg/ml) of control or FLAG-USP13 transfected HEK293T cells for indicated time points. β-actin is a loading control. **E** Immunoblot of USP13 and MYC in transfected HEK293T cells upon treatment with either DMSO or indicated concentrations of Spautin-1 for 24 h. **F** Schematic diagram showing the domain organization of USP13 with deletion constructs used. **G** Immunoblot analysis of HEK293T cells transfected with Myc-tagged WT or deletion mutants of USP13. Lysates were blotted by anti-Myc-tag and anti-MYC antibodies. Band intensities were quantified and normalized using β-actin levels. **H** Lysates from HEK293T cells expressing the indicated plasmids treated with MG132 (10 μM) for 6 hr were subjected to IP for FLAG-tagged MYC and then immunoblotting for HA-tagged ubiquitin. Total cell lysates (TCL) correspond to 10% of the total protein amount used for the precipitation. **I** The ∆Znf∆UBA mutant form of USP13 fails to deubiquitinate MYC. **J** USP13 knockdown (KD) increased the ubiquitin level of MYC. HEK293T cells (shNS, KD1, and KD2) were transfected with FLAG-MYC and HA-Ub. **K** HEK293T cells were transfected with FLAG-MYC, HA-Ub (WT, K48, or K63), and Myc-tagged USP13

Given that USP13 regulates the stability of MYC in glioblastoma and hepatocellular carcinoma, contributing to self-renewal or tumorigenic potential [31, 51], we hypothesized that USP13 would be directly associated with MYC protein abundance in lung cancer. Overexpression of USP13 significantly increased the expression of MYC at the protein level but not at the mRNA level, indicating post-translational regulation of MYC by USP13 (Fig. 5A and Fig. S5A). Exogenous MYC expression levels were significantly decreased by *USP13* knockdown (Fig. 5B). Co-immunoprecipitation showed the interaction of USP13 with MYC (Fig. 5C). In the cycloheximide (CHX) chase assay, exogenous USP13 overexpression enhanced the stability of MYC protein (Fig. 5D).

To investigate the impact of USP13's deubiquitinating enzyme activity in stabilizing MYC, we first examined the level of FLAG-MYC in HEK293T cells treated with Spautin-1, a small molecule inhibitor of the deubiquitinase activity of USP10/USP13 [52]. Inhibition of USP13 resulted in a reduction in FLAG-MYC protein abundance (Fig. 5E). It is intriguing to note that treatment with Spautin-1 also led to a decrease in USP13 level. USP13 has delineated functional domains, namely, a zinc finger domain (ZnF) and two proximal ubiquitin-binding domains (UBA1/2) (Fig. 5F) [53]. We sought to determine which, if any, of these domains were essential for the stabilization of MYC. The deletion of either the ZnF or UBA1/2 domain of USP13 resulted in a decrease in its ability to increase MYC protein, whereas the deletion of both domains completely eliminated its ability to upregulate MYC protein levels (Fig. 5G).

Overexpression of wild-type USP13 decreased the ubiquitin-conjugated MYC level (Fig. 5H). However, mutant USP13^∆Znf∆UBA^ did not have the same effect (Fig. 5H and I). In contrast, the knockdown of USP13 increased the ubiquitin levels of MYC (Fig. 5J). To further investigate the chain specificity of MYC deubiquitylation by USP13, we co-expressed USP13 with HA-tagged ubiquitin carrying a single lysine residue at positions K48, K63, or K48R. Immunoblotting data showed that USP13 acted on the K48-linked ubiquitin chain on MYC, not K63 (Fig. 5K). The K48R mutation abolished USP13 from removing the ubiquitin molecule from MYC (Fig. S5B). These results indicate that USP13 increases MYC protein stability by cleaving the K48-linked ubiquitin conjugation of MYC.

### Club cells are the origin of LUSC in the KPU model

**Fig. 6:**
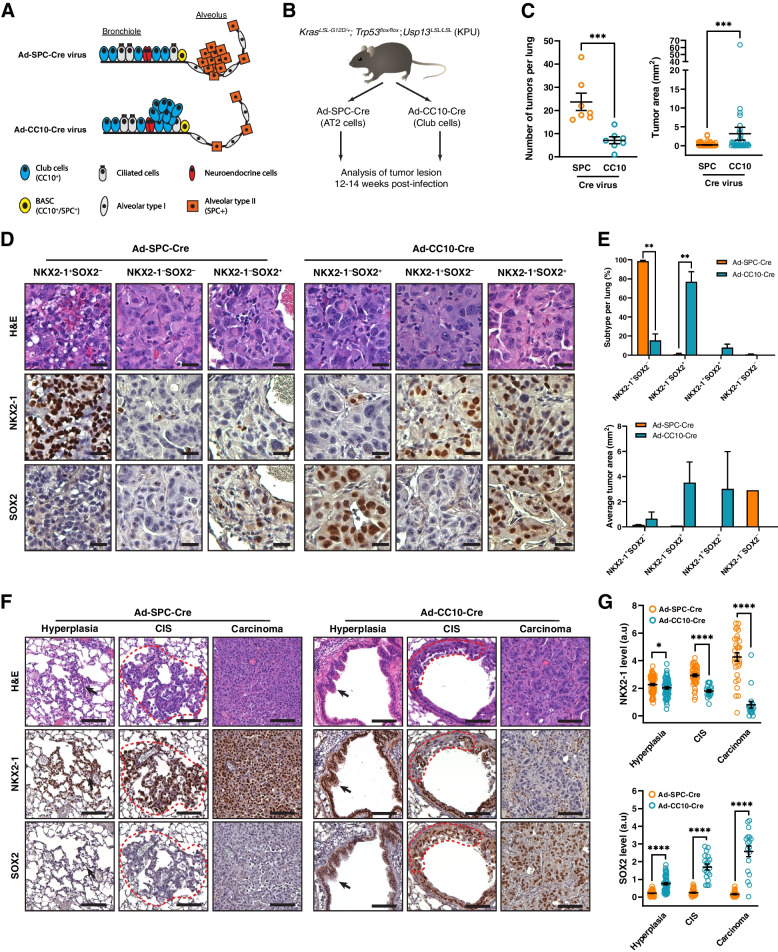
LUSC originates from CC10^+^ club cells in the KPU model. **A** Schematic of two origins of lung adenocarcinoma. Cre recombinase expression was restricted to specific cell types in the adult lung by cell type-restricted Ad-Cre viruses; Cre recombinase is expressed in alveolar type II cells (orange) and club cells (blue) by Ad5-SPC-Cre and Ad5-CC10-Cre, respectively. Bronchioalveolar stem cells (BASCs, yellow) can express Cre recombinase by both viruses **B** Schematic of the experimental procedure using Ad5-SPC-Cre and Ad5-CC10-Cre viruses. KPU mice were infected with cell-type restricted Ad-Cre viruses via intratracheal administration. **C** Quantification of individual tumor number (left) and area (right) in KPU mice at 12-14 weeks post-virus infection (*n=*7 mice/group). **D** Representative images of H&E, NKX2-1, and SOX2 staining from lung tumors with indicated Cre infection. Scale bar = 25 µm **E** Quantification of proportion (top) and size (bottom) of lesions as indicated in **D F** Expression of NKX2-1 and SOX2 during cancer development in KPU mice infected with Ad5-SPC-Cre or Ad5-CC10-Cre. Arrow and dotted lines indicate hyperplasia and carcinoma *in situ* (CIS), respectively. Scale bar = 50 µm **G** IHC quantification for NKX2-1 (top) and SOX2 (bottom). Each dot represents one tumor nodule from seven mice per group. a.u., arbitrary unit. In **C**, **E**, and **G**, error bars indicate mean ± SEM. Two-tailed unpaired t-tests, ns = not significant, *p < 0.05, **p < 0.01, ***p < 0.001, ****p < 0.0001

In normal murine lungs, AT2 cells (SPC^+^) are NKX2-1-positive but SOX2-negative, while club cells (CC10^+^) at the bronchioles are double positive for NKX2-1 and SOX2 (Fig. S6A and B). To investigate the effect of lung progenitors in KPU-driven mixed LUAD and LUSC tumor subtypes, we induced restricted expression of Cre recombinase in club cells and AT2 cells via intratracheal delivery of Ad5-CC10-Cre or Ad5-SPC-Cre adenovirus to KPU mice, respectively (Fig. 6A and B). The tumor burdens and histopathology of both groups of KPU mice were examined 12-14 weeks post-infection. AT2 cells targeting Ad5-SPC-Cre-infected KPU lungs showed an increase in the number of lung tumor nodules but were small in size and revealed the presence of adenocarcinoma histology. When KPU mice were infected with the CC10-Cre virus in the club cells, they developed notably larger tumors with squamous histology features (Fig. 6C and Fig. S6C). We then analyzed the expression of the lung cancer lineage specifiers NKX2-1 and SOX2. Ad5-SPC-Cre-infected KPU tumors were mostly positive for NKX2-1 but negative for SOX2, with only a small number of tumors showing negative staining for NKX2-1 (Fig. 6D and E). The majority of tumors in KPU lungs by Ad5-CC10-Cre were identified as NKX2-1-negative and SOX2-positive (NKX2-1^–^/SOX2^+^), although a few tumors exhibited NKX2-1^+^/SOX2^–^ or NKX2-1^+^/SOX2^+^ characteristics (Fig. 6D and E). Next, we examined how those factors are expressed during the tumor development in KPU mice after being induced by either Ad5-SPC-Cre or Ad5-CC10-Cre viruses. Tumors originating from AT2 cells continuously showed an NKX2-1^+^/SOX2^–^ pattern from early hyperplastic lesions to invasive carcinoma (Fig. 6F, left). Following CC10-Cre infection in the KPU lung, hyperplasia at bronchioles initially exhibited positive expression for both factors; however, NKX2-1 expression was subsequently downregulated at the carcinoma *in situ* (CIS) stage and remained consistently negative in invasive carcinoma (Fig. 6F, right). NKX2-1 levels were dramatically decreased, while SOX2 levels were increased in Ad5-CC10-Cre-infected KPU lungs (Fig. 6G). These data suggest that squamous tumors in KPU mice originate from club cells, not AT2 cells. Additionally, USP13 might contribute to the downregulation of NKX2-1 during the early stages of LUSC development.

### USP13 suppresses NKX2-1 while upregulating SOX2 in club cell-originated LUSC development

**Fig. 7:**
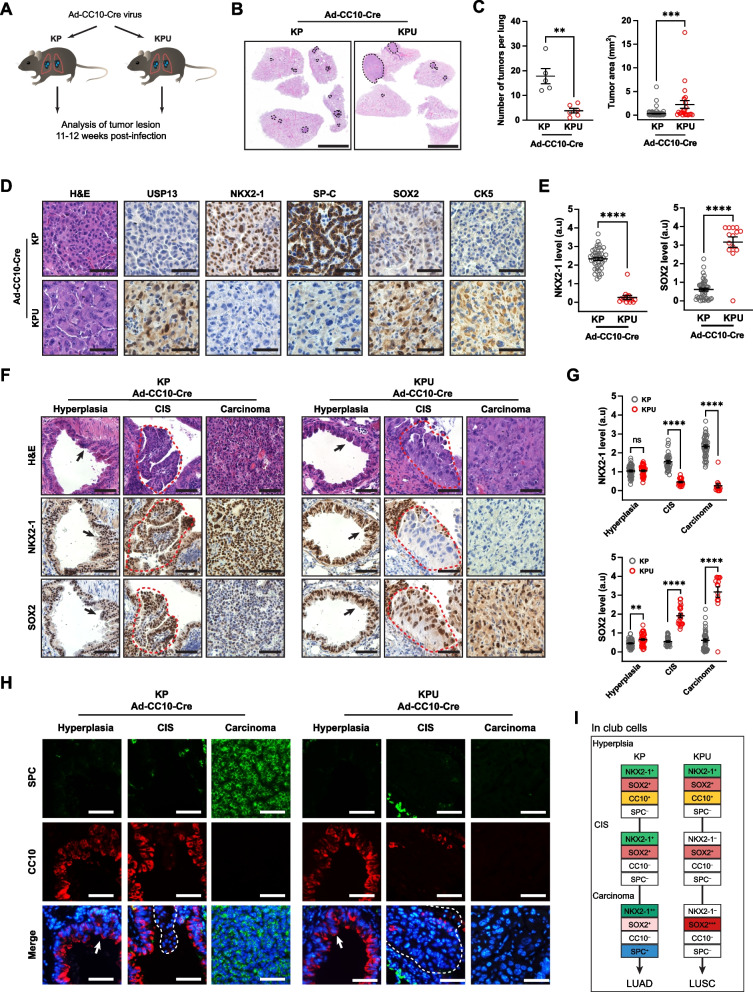
Enhanced USP13 expression switches lineage factor expression in KP-transformed club cells. **A** Experiment design: KP or KPU mice received a single intratracheal injection of cell-type-restricted Ad5-CC10-Cre virus. The experiment was terminated 11-12 weeks post-viral infection. **B** Representative hematoxylin and eosin (H&E) staining of KP and KPU lung at 12 weeks post-Ad5-CC10-Cre infection. Dotted line indicates a tumor nodule. Scale bar = 5 mm. **C** Quantification of individual tumor number (left) and area (right) in KP and KPU mice at 11-12 weeks post-virus infection (*n=*5 mice/group). **D** Representative images for H&E, USP13, NKX2-1, SPC, SOX2, and CK5 IHC stains of the KP and KPU tumors post-Ad5-CC10-Cre infection. Scale bar = 50 µm. **E** Quantification of NKX2-1 (left) and SOX2 (right) levels in KP and KPU tumors. **F** Expression of NKX2-1 and SOX2 during cancer progression in KP and KPU mice following Ad5-CC10-Cre infection. Hyperplasia (arrow) and carcinoma *in situ* (CIS) (dotted line) are indicated. Scale bar = 50 µm. **G** IHC quantification for NKX2-1 (top) and SOX2 (bottom). Each dot represents one tumor nodule. a.u., arbitrary unit. **H** SPC and CC10 expression during cancer progression in Ad5-CC10-Cre infected KP and KPU lungs. Hyperplasia (arrow) and carcinoma *in situ* (CIS) (dotted line) are indicated. Scale bar = 50 µm. (**I**) Schematic summarizing the expressional change of lineage factors and markers in KP and KPU club cells (CC10^+^) during lung cancer development. In (**C**), (**E**), (**G**), error bars indicate mean ± SEM. Two-tailed unpaired t-tests, ns = not significant, **p < 0.01, ***p < 0.001, ****p < 0.0001

Both AT2 cells and club cells are known as cells of origin for KP-induced LUAD development [54, 55]. In previous studies on KP GEMMs, it was found that bronchiolar hyperplasia and adenoma derived from CC10^+^ club cells showed positive staining for SOX2, but SOX2 expression was downregulated in adenocarcinoma, leading to LUAD lineage identity [55–57]. In the KPU model, USP13 overexpression in club cells led to squamous cell carcinoma development in the KP genetic alteration background. Therefore, we further examined the tumor progression of KP and KPU models after delivering Ad5-CC10-Cre or Ad5-SPC-Cre virus, respectively (Fig. 7A, Fig. S7A). Similar to Ad5-CMV-Cre-infected KP and KPU mice (Fig. 2), Ad5-CC10-Cre-induced KPU lungs developed a small number of tumor lesions but large tumors compared to KP lungs (Fig. 7B and C). As expected, club cell-originated KP tumors showed adenocarcinoma histology with positive expression of LUAD markers (NKX2-1 and SPC) (Fig. 7D). In contrast, KPU tumors were negative for LUAD markers but positive for squamous cell markers such as SOX2 and CK5. Interestingly, the AT2 cell-originated KPU tumors developed a much smaller number of tumor lesions with smaller tumor sizes than AT2 cell-originated KP adenocarcinomas (Fig. S7B and C). Both KP and KPU lesions following Ad5-SPC-Cre infections exhibited typical LUAD marker (NKX2-1 and SPC)-positive expression, with adenocarcinoma histology (Fig. S7D).

In KP and KPU tumors following Ad5-CC10-Cre infection, we classified subtypes of KP and KPU tumors by NKX2-1 and SOX2 expression levels (Fig. S7E and F). NKX2-1^–^SOX2^+^ tumors were predominantly observed in the CC10-Cre-infected KPU (Fig. S7F). KPU tumors had ~5.2-fold higher expression levels of SOX2 than KP tumors (*p <* 0.0001) (Fig. 7E). Interestingly, CC10-Cre-induced KP tumors also showed SOX2-positive expression, and its level was heterogeneous (Fig. 7E and E). Two subtypes (NKX2-1^+^SOX2^–^ and NKX2-1^+^SOX2^+^) exhibited similar proportions in CC10-Cre-induced KP lungs (Fig. S7F). In Ad5-CC10-Cre-infected KP and KPU lungs, hyperplastic cells at bronchioles in KP and KPU were stained positive for both NKX2-1 and SOX2 (Fig. 7F). Our data demonstrate that KP-driven hyperplastic lesions and adenomas transformed from club cells are positive for SOX2, but SOX2 expression becomes significantly decreased and partially lost in more advanced lesions (Fig. 7F and G). During the CIS stage, there were noticeable differences in the expression patterns of NKX2-1 and SOX2 between the lungs of KP and KPU mice. In contrast to the KP model, NKX2-1 expression was dramatically decreased at the CIS stage in KPU models, while SOX2 levels were high throughout LUSC development (Fig. 7F and G).

We next examined the expression of the cell identity markers CC10 and SPC during tumor development in KP and KPU mice after Ad5-CC10-Cre induction (Fig. 7H). Hyperplastic lesions in the bronchioles of both KP and KPU showed CC10^+^/SPC^–^ staining. Then, KP-transformed club cells underwent stepwise lineage marker conversion from CC10^+^/SPC^–^ to CC10^–^/SPC^+^ from hyperplastic lesions to adenomas and invasive adenocarcinoma (Fig. 7H). In contrast, KPU carcinomas did not show elevated SPC expression, displaying different lineage marker conversions of CC10^+^/SPC^–^ to CC10^–^/SPC^–^ (Fig. 7H). In both KP and KPU models, hyperplasia, CIS, and adenocarcinoma induced by Ad5-SPC-Cre showed AT2 cell-like lineage marker expression (CC10^–^/SPC^+^) (Fig. S7G). These data suggest that USP13 is involved in cell lineage reprogramming and switching the fate of oncogenic KRAS-mediated transformed CC10^+^/SPC^–^ club cells to CC10^–^/SPC^–^, which leads to squamous cell differentiation instead of adenocarcinoma progression (Fig. 7I).

### The elevation of MYC protein levels by USP13 contributes to promoting squamous carcinoma features

**Fig. 8:**
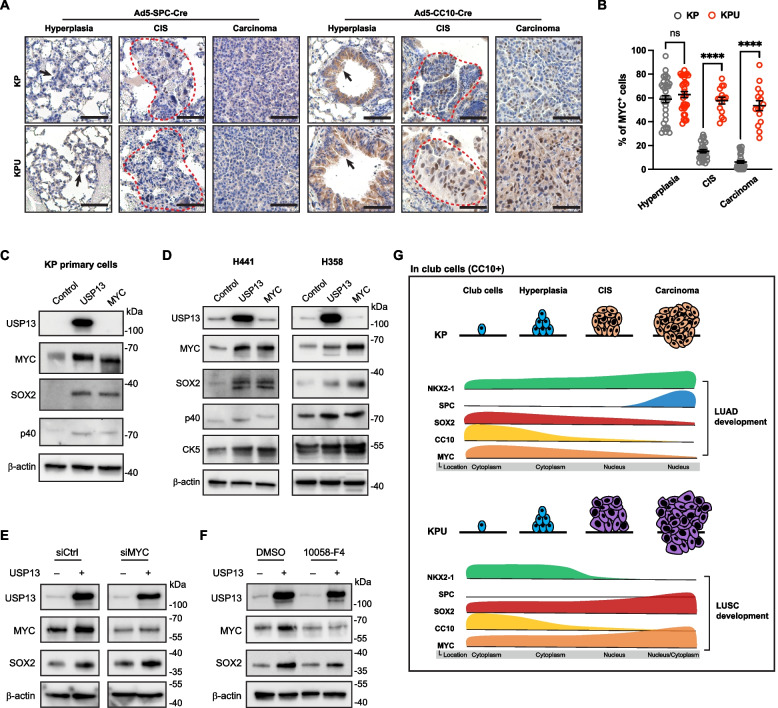
USP13 elevates MYC expression in lung cancer, contributing to the elevation of LUSC features. **A** MYC expression during cancer progression in KP and KPU mice following Ad5-SPC-Cre (left) or CC10-Cre (right) infection. Hyperplasia (arrow) and carcinoma *in situ* (CIS) (dotted line) are indicated. Scale bar = 50 µm. **B** IHC quantification for the portion of MYC^+^ cells in the lesions. Each dot represents one tumor nodule. Error bars indicate mean ± SEM. Two-tailed unpaired t-tests, ns = not significant, ****p < 0.0001. **C** Western blot of USP13, MYC, SOX2, p40, and β-actin on mouse KP cell line expressing exogenous USP13 or MYC. **D** Western blot showing USP13, MYC, SOX2, p40, CK5, and β-actin in human LUAD cell lines expressing exogenous USP13 or MYC. **E** Western blot of USP13, MYC, SOX2, and β-actin in H441 cells with knockdown of MYC using siRNA followed by transfected with mock or USP13. **F** Western blot of USP13, MYC, SOX2, and β-actin in H441 cells treated with either DMSO or 10 μM of 10058-F4 (MYC inhibitor) for 24 h followed by transfection with mock or USP13. **G** Schematic showing the proposed model of how USP13 promotes LUSC tumorigenesis in club cells

5Given the strong connections between USP13 and MYC in human LUSC and KPU lung squamous tumors (Fig. 4) and the stabilization of MYC protein through the deubiquitinating activity of USP13 (Fig. 5), we examined the expression pattern of MYC during KP and KPU tumorigenesis following Ad5-SPC-Cre or Ad5-CC10-Cre infection. Ad5-SPC-Cre-derived KP and KPU tumors showed minimal detection of the MYC protein, regardless of USP13 overexpression (Fig. S8A, left). Elevated MYC expression was observed in both KP and KPU tumors derived from CC10^+^ cells (Fig. S8A, right). KP tumors showed positive but weaker MYC expression than KPU tumors (Fig. S8B and C). Strong expression of MYC was detected in both the cytoplasm and nucleus of invasive squamous carcinoma in KPU lungs (Fig. 8D). In the CIS stage of Ad5-CC10-Cre-infected KP lungs, the portion of MYC^+^ cells was drastically reduced and continued to decrease during adenocarcinoma tumorigenesis (Fig. 8A and B). In humans, MYC has been reported to be predominantly in the cytoplasm in the normal basal cells of the airways of patients with lung cancer, but cytoplasmic MYC is transferred into the nuclei of premalignant lesions and squamous cells [22]. Similarly, in Ad5-CC10-Cre-infected KPU mice, MYC was translocated into the nucleus at the squamous CIS lesion, and the strong nuclear expression of MYC persisted during squamous carcinoma development (Fig. 8A, Fig. S8E). These findings suggest that USP13's deubiquitinating activity might stabilize MYC protein during the early stage of lung squamous tumorigenesis and that this effect is limited to squamous tumors originating from club cells.

A recent study proposed MYC as a putative molecular driver linked to LUAD to LUSC histological transformation [58]. To determine whether USP13 can alter the lineage characteristics of advanced lung tumors through MYC, we overexpressed USP13 in the murine adenocarcinoma KP primary cell line and human LUAD cell lines followed by immunoblotting for squamous markers, such as SOX2, p40, or CK5. UPS13 overexpression alone induced the expression of squamous markers (Fig. 8C and D). In addition, MYC overexpression also increased the level of squamous markers (Fig. 8C and D), implying that USP13 can induce MYC and squamous cell lineage programs in murine and human LUAD cells and that MYC is sufficient to enhance LUSC characteristics. To determine whether MYC is necessary for USP13 to upregulate SOX2 expression, we knocked down or inhibited MYC and then overexpressed USP13 in H441 cells. Despite MYC knockdown, the overexpression of USP13 still resulted in an increase in SOX2 levels in H441 cells (Fig. 8E). In addition, USP13 still upregulated the expression of SOX2 under inhibition of MYC using a small-molecule c-MYC inhibitor, 10058-F4 treatment (Fig. 8F), suggesting that USP13 could be associated with squamous cell markers in a MYC-dependent manner and MYC-independent manner. Based on these findings, it appears that overexpression of USP13 causes an increase in MYC protein during the initial stages of tumor development originating from club cells, which may contribute to the development of LUSC. Moreover, we revealed that overexpression of USP13 and/or MYC induces squamous cell lineage markers in human and murine LUAD.

## Discussion

In this study, we found that USP13 is involved in shifting lung club cell fate in the context of KP genetic alteration by promoting lung lineage reprogramming and MYC upregulation, which leads to LUSC tumorigenesis. USP13 overexpression suppresses NKX2-1 expression while increasing SOX2 at the initial stages of lung tumorigenesis, suggesting a role for USP13 in lung club cell lineage reprogramming for LUSC development (Fig. 8G). In addition, strong nuclear MYC expression throughout squamous cell carcinoma is a critical feature for KPU-driven LUSC (Fig. 8G).

The inhibition of TP53 signaling and the activation of MYC have been identified as important molecular characteristics in early LUSC carcinogenesis in humans [22]. *TP53* mutations are more prevalent in LUSC than LUAD [59, 60]. Lineage plasticity is associated with the loss of tumor suppressor genes such as *RB1*, *PTEN*, and *TP53* [6]. The combined loss of *RB1* and *TP53* promotes lineage plasticity and transdifferentiation in prostate and lung cancer in mouse models [61–63]. However, the loss of tumor suppressor genes appears insufficient to drive lineage plasticity in the context of the lung. *RB1* and *TP53* abrogation is not enough to promote a neuroendocrine feature in lung cancer cells [64]. Another study demonstrated that most LUAD patients with concurrent *RB1* and *TP53* alterations do not undergo histological transformation into SCLC [65]. p53 loss in club cells decreased ciliated cell differentiation and increased self-renewal and proliferative capacity [66]. Moreover, club cells have the capacity to produce basal-like cells *in vitro* and, *in vivo*, give rise to bronchioalveolar stem cells as well as ciliated cells following p53 loss. Unlike KPU, which resulted in 100% LUSC tumor development, KU mice only developed LUAD tumors, similar to the K mice (Fig. S2F). This suggests a potential interaction between USP13 and TP53 signaling in lineage plasticity, and the functional alteration of TP53 could be crucial in LUSC progression mediated by USP13.

Recently, it has been reported that USP13-mediated deubiquitination is associated with MYC in cholangiocarcinoma [67], glioma stem cells [31], and hepatocellular carcinoma [51]. We identified critical domains of USP13 important for stabilizing MYC, and USP13 acts on K48-mediated polyubiquitination on MYC (Fig. 5). In addition to MYC stabilization, we also observed distinctive nuclear translocation of MYC in the CIS lesion from Ad5-CC10-Cre-infected KPU club cells, and its high level was maintained in invasive squamous cell carcinoma, suggesting enhanced MYC activity during KPU-driven LUSC tumorigenesis. Enhanced MYC target gene expression together with a concomitant increase in the nuclear translocation of MYC were detected in human premalignant lesions and LUSC tumors [22]. This provides strong evidence that enhanced MYC activity is associated with lung squamous carcinogenesis, even in the absence of a significant upregulation in MYC mRNA expression [22]. Intriguingly, in a *Kras*^*G12D*^ activation in combination with deletion of F-box/WD repeat-containing protein 7 (*Fbxw7*) murine model, CC10^+^ bronchiolar club cells give rise to LUSC but not from the tracheal basal cells and alveolar AT2 cells [26]. *FBXW7* encodes a component of the SCF ubiquitin E3 ligase complex involved in ubiquitination and proteasome degradation of oncoproteins, including MYC, Cyclin E, c-JUN, and NOTCH1 [68, 69]. Collectively, these findings suggest that concurrent with an appropriate set of oncogenic stimuli, the molecular interaction among USP13-mediated deubiquitination, increased MYC activity, and p53 functional alteration may play a critical role in squamous differentiation from oncogenic transformed cells of origin. Moreover, ubiquitin signaling could be directly associated with LUSC tumorigenesis by involving lineage reprogramming to the squamous cell carcinoma program.

Previous murine models have revealed that SOX2 overexpression is necessary but not sufficient for lineage switching. To induce tumors with squamous histology in multiple cells of origin in the lung, SOX2 overexpression needs to collaborate with loss of NKX2-1 together with other oncogenic stimuli or loss of multiple tumor suppressor genes, including *Pten*, *Cdkn1a*, *Cdkn2a/b*, *Lkb1*, *Keap1*, *Trp53*, and *Nkx2-1* [48, 70–76]. Activation of the *Kras* mutant in mouse models lacking *Nkx2-1* results in the loss of LUAD features and the subsequent acquisition of gut-related traits [16, 74, 77, 78]. *FoxA1/2*, another lineage-specifying transcription factor, drives gastric differentiation and suppresses squamous identity in NKX2-1-negative lung cancer [49, 79]. Loss of *Nkx2-1* and overexpression of *Sox2* was sufficient to generate squamous differentiation [74]. Our KPU model demonstrates that the deubiquitinating enzyme USP13 can switch on squamous carcinogenesis in club cells without direct genetic engineering of key lineage-defining factor genes, such as *Sox2*, *Nkx2-1, and FoxA1/2.* Interestingly, KPU-derived LUSC tumorigenesis showed a distinctive loss of NKX2-1 and increased SOX2, demonstrating that USP13 is involved in cell lineage reprogramming at the initial stage of LUSC development. Therefore, in the oncogenic KRAS-mediated transformed club cells, USP13 seems to prevent those club cells from adopting an AT2-like adenocarcinoma identity, instead switching the fate of cells to squamous cell traits. USP13 directly deubiquitinates and upregulates MYC protein stability (Fig. 5), and MYC overexpression alone upregulated SOX2 in NSCLC cell lines (Fig. 8). Therefore, it is possible that USP13 is associated with SOX2 and NKX2-1 through MYC stabilization at the early stage of LUSC development. Future molecular mechanistic studies are required to elucidate whether USP13 is directly involved in the interplay between NKX2-1 and SOX2 and how USP13-mediated deubiquitination promotes lung lineage plasticity reprogramming.

Recently, the histological transformation (transdifferentiation) of LUAD to LUSC or SCLC has emerged as a resistance mechanism to tyrosine kinase inhibitor (TKI)-targeted therapy or chemotherapy in clinics [6, 64, 80–84]. Squamous transformation has also been described in *KRAS*^*G12C*^-mutated LUAD as a mechanism of resistance to targeted therapy for KRAS mutation [85]. Liver kinase B1 (Lkb1) has been proposed as a molecular driver of LUAD to LUSC transdifferentiation using a mouse model [86, 87]. The *Kras*^*G12D*^-driven LUAD mouse model can progress to LUSC upon subsequent deletion of *Lkb1*. Inactivation of *Lkb1* and *Pten* in the mouse lung leads to LUSC features [88]. However, due to the scarcity of well-annotated paired pre- and post-clinical samples and the lack of preclinical models, the molecular drivers underlying LUAD to LUSC transdifferentiation remain largely unknown. Our findings in this study suggest that the upregulation of USP13 might contribute to LUAD to LUSC transdifferentiation. Chromosome 3q amplification was found in squamous transformation tissues in osimertinib-resistant *EGFR* mutant lung cancers [83]. Quintanal-Villalonga *et al*. identified that lineage plasticity mechanisms leading to the histological transformation of human EGFR-mutant LUAD have been linked to the MYC, NOTCH, Hedgehog, PI3K/AKT, and WNT pathways [6, 58]. These pathways were enriched in KPU tumors (Fig. 3). KPU tumors displayed significant heterogeneity, leading to their categorization into two distinct clusters, KPU type 1 and type 2 (Fig 3A). Both clusters showed LUSC signature gene expression. In particular, KPU type 1 demonstrated heightened SCLC-like traits along with LUSC characteristics (Fig 3B). Although different signaling pathways were enriched in each cluster (Fig 3E), Hedgehog, Notch, and PI3K/Akt/mTOR signaling play an important role in the development of LUSC and SCLC [89–91]. Molecular heterogeneity in KPU tumors can be caused by different mechanisms such as high plasticity of club cells and tumor microenvironment [92, 93]. Moreover, the USP13-mediated deubiquitination process directly upregulated MYC protein levels with increased expression of SOX2 and squamous features in KP mouse and human lung adenocarcinoma cells. MYC has been linked to histological transdifferentiation in SCLC and pancreatic cancer, suggesting a role in tumor lineage plasticity [47, 94]. These findings suggest that the shared molecular networks and dysregulated pathways might be involved in USP13-mediated LUSC de novo development and LUAD-to-LUSC transdifferentiation. Additionally, it will be intriguing to determine whether the subsequent overexpression of USP13 from oncogenic-mutant LUAD can lead to LUSC transdifferentiation.

## Conclusions

In summary, our novel mouse model showed that USP13 switches on squamous carcinogenesis from club cells, and USP13-mediated lineage reprogramming is crucially defined in the cell of origin and genetic background. USP13-mediated deubiquitination leads to MYC upregulation, which may provide a novel molecular mechanism for LUSC development and LUSC transdifferentiation. Future studies are warranted to determine how USP13 reprograms lineage plasticity in USP13-amplified lung cancers and whether targeting USP13 can overcome therapeutic-resistant LUSC-transformed lung tumors.

### Supplementary Information

**Additional file 1: Table S1**.

**Additional file 2: Table S2**.

**Additional file 3: Table S3**.

**Additional file 4: Table S4**.

**Additional file 5: Fig. S1. **(A) Genomic alterations of USP family members in lung squamous cell carcinoma determined by cBioPortal analysis of TCGA databases (*n=*469). (B) Illustration of samples with 3q26 amplification in 469 LUSC patients. The 3q distal regions have been magnified to show the position of *USP13*, *SOX2, *and *TP63 *genes. (C) Correlation between copy number variation of *USP13 *and mRNA expression of squamous markers according to *KRAS *mutation in LUAD. Squamous markers include keratin 16 (*KRT16*), keratin 17 (*KRT17*), uroplakin 1B (*UPK1B*), and arachidonate lipoxygenase 3 (*ALOXE3*). X-axis is copy number variation of *USP13 *and the y-axis is log2(x+1) transformed RSEM normalized count. Error bars indicate mean ± SEM. Two-tailed unpaired t-tests, *p < 0.05, **p < 0.01. **Fig. S2. **(A) Schematic of *KrasLSL-G12D/+*; *Trp53flox/flox* (KP) and *KrasLSL-G12D/+*; *Trp53flox/flox*; *Usp13LSL/LSL* (KPU) alleles. (B) KP tumors were characterized by glandular formation (representative LUAD feature). The bottom is a high magnification of the top. Scale bar = 100 μm. (C) Different histological features in KPU tumors. (a) KPU tumors showed squamous characters such as keratin material (asterisk) and intercellular bridge (arrow). A small portion of the tumor exhibited small cell lung carcinoma (SCLC) (b) or carcinosarcoma-like histology (c).(a’), (b’) and (c’) are higher magnifications of the boxed area in (a), (b), and (c), respectively. Scale bar = 100 μm (D) H&E staining of KPU mouse lung from Figure 2C (left). Black and red lines indicate LUAD and LUSC lesions, respectively. Scale bar = 5 mm. Representative images for H&E, USP13, NKX2-1, SPC, and SOX2 IHC stains of the LUAD component in KPU tumors (right). Scale bar = 50 μm. (E) Schematic of *KrasLSL-G12D/+* (K) and *KrasLSL-G12D/+*; *Usp13LSL/LSL* (KU) alleles. (F) Quantifying individual tumor number and area in K and KU mice at 55-58 weeks post-Ad-CMV-Cre infection (*n*= 3 and n= 5, respectively). Error bars indicate mean ± SEM. Two-tailed unpaired t-tests, *p < 0.05, **p < 0.01, ***p < 0.01. (G) Representative images for H&E, USP13, NKX2-1, SPC, SOX2 IHC stains of K and KU tumors. Scale bar = 50 μm. **Fig. S3.** (A) Heatmap showing the expression of marker genes for LUAD, LUSC, and SCLC in TCGA LUAD and LUSC samples. (B) Heatmap visualization of differentially expressed genes (DEGs) (log2FC > 3.5, *p* < 0.01, and q < 0.01) between KP and KPU tumor samples. (C) Enrichment plots for hallmark xenobiotic metabolism, KRAS signaling down, and peroxisome for KP and KPU tumors. (D) GSEA analysis for hallmark G2M checkpoint, E2F targets, and MYC targets version 1 for LUAD and LUSC. (E) IPA analysis shows the top ten canonical pathways enriched in LUSC compared with LUAD. Each bar's color indicates predicted pathway activation or inhibition; Orange, positive z-score (activation); white, zero z-score; blue, negative z-score (inhibition); gray, no pattern. (F) Upstream regulator analysis exhibits transcription factors' predicted activation status in human LUSC. A positive z-score indicates activation and a negative z-score indicates inhibition. **Fig. S4.** (A) Correlation between *USP13* and *MYC* mRNA expression (left), *USP13* and *SOX2* mRNA expression (center), and *MYC* and *SOX2* mRNA expression (right) in LUSC. Data was obtained from cBioportal (TCGA, PanCancer Atlas) (*n*= 469). mRNA Expression is log2(x+1) transformed RSEM normalized count. (B) Correlation between *USP13* and *MYC* mRNA expression in LUAD. Data was obtained from cBioportal (TCGA, PanCancer Atlas) (*n*= 507). (C-E) Representative IHC-staining images of USP13 (C), MYC (D), and SOX2 (E) in LUAD (left) and LUSC (right). The red boxed areas on the left images were magnified and shown on the right. Black scale bar = 100 μm, Greyscale bar = 50 μm. (F) Correlation between USP13 and MYC in the cytoplasm (left) and nucleus (right) of NSCLC patient samples. (G) Correlation between USP13 and SOX2 expression (left) and MYC and SOX2 expression (right) in LUSC. Data was obtained from Figure 4B (*n*= 130). IHC scores are analyzed using a two-tailed Pearson correlation coefficient. (H) Low magnification images of Figure 4F. Scale bar = 200 μm. **Fig. S5. **(A) Quantification of MYC expression levels relative to the vehicle is shown (left). qPCR of *USP13* and *MYC* mRNA expression in HEK293T cells with or without USP13 overexpression. mRNA level was normalized to β-actin. Data are shown as means ± SD. ns, not significant, **p < 0.01, and ****p < 0.0001 (unpaired two-tailed t-test). (B) USP13-mediated deubiquitylation acts upon the Lys48 (K48) ubiquitination of MYC. 293T cells were transfected with FLAG-MYC, HA-Ub (WT or K48R), and Myc-tagged USP13. **Fig. S6. **(A) Section of wild-type mouse lung stained with anti-SPC (green) and anti-CC10 (red). Scale bar = 50 μm. (B) Section of wild-type mouse lung stained with anti-NKX2-1 (green) and anti-SOX2 (red). Scale bar = 50 μm. (C) Representative hematoxylin and eosin (H&E) staining of KPU lung infected by Ad5-SPC-Cre or Ad5-CC10-Cre virus. Dotted lines indicate tumor nodules. Scale bar = 5 mm. **Fig. S7.** (A) Experiment design: KP or KPU mice received a single intratracheal injection of celltype- restricted Ad-SPC-Cre virus. The experiment was terminated 11-12 weeks post-viral infection. (B) Representative hematoxylin and eosin (H&E) staining of KP and KPU lung at 12 weeks post-Ad-SPC-Cre infection. Dotted line indicates a tumor nodule. Scale bar = 5 mm. (C) Quantification of individual tumor number (left) and area (right) in KP and KPU mice at 11-12 weeks post-Ad-SPC-Cre virus infection (*n*= 5 mice/group). (D) Representative images for H&E, USP13, NKX2-1, SPC, and SOX2 IHC stains of the KP and KPU tumors. Scale bar = 50 μm. (E) Representative NKX2-1 and SOX2 staining images from lung tumors in Ad-CC10-Cre infectedKP (left) and KPU (right) mice. Scale bar = 50 μm. (F) Quantification of lesions with NKX2-1 and SOX2 expression patterns from (E). (G) SPC and CC10 expression during cancer progression inAd-SPC-Cre infected KP and KPU lungs. Hyperplasia (arrow) and carcinoma *in situ* (CIS) (dotted line) are indicated. Scale bar = 50 μm. In (C) and (F), error bars indicate mean ± SEM. Two-tailed unpaired t-tests, *p < 0.05, **p < 0.01. **Fig. S8.** (A) Representative images for IHC staining of MYC in the KP and KPU tumors post- Ad5-SPC-Cre (left) or Ad5-CC10-Cre (right) infection. Scale bar = 50 μm (B) IHC quantification for MYC in tumors. Error bars indicate mean ± SEM. Two-tailed unpaired t-test, ns = not significant, ****p < 0.0001. (C and D) Representative images of KP (C) and KPU (D) tumors with low, intermediate, and high MYC expression. Scale bar = 50 μm. (E) Cellular localization of MYC during tumor progression in KP (left) and KPU (right) lungs following Ad5-CC10-Cre infection. The bottom figures are higher magnifications of the boxed area in the top figures. Arrows indicate the cellular location of MYC protein; Black, cytoplasm; Green, whole cell; Orange, nucleus; Cyan, whole cell with nuclear enrichment. Black scale bar = 50 μm, white scale bar = 25 μm.

## Data Availability

All software is commercially available or cited in previous publications. RNA sequencing data in this study is deposited in NCBI GEO (https://www.ncbi.nlm.nih.gov/geo/) and is publicly available as the date of publication under accession GSE244847. TCGA lung adenocarcinoma and squamous cell carcinoma gene expression data were obtained from the GDC data portal (https://portal.gdc.cancer.gov/).
