# Supplementary material for: USP13 drives lung squamous cell carcinoma by switching lung club cell lineage plasticity
Source: Mol Cancer. 2023 Dec 13;22:204. doi: 10.1186/s12943-023-01892-x (PMC10717271; doi:10.1186/s12943-023-01892-x)
Supplement: Supplementary file 1 — Additional file 1: Table S1. [file 12943_2023_1892_MOESM1_ESM.pdf]

Genotyping primers			
**Gene**	**Primer sequence (5' → 3')**	**PCR condition**	**Product size**
Kras$^{G12D/+}$	WT_FW: TGTCTTTCCCCAGCACAGT<br>Mutant_FW: GCAGGTCGAGGGACCTAATA<br>Common_RV: CTGCATAGTACGCTATACCCTGT	95 ℃ for 30 s, 65 ℃ for 15 s (-0.5 ℃ per cycle decrease), 68 ℃ for 10 s, for 10 cycles (touch down), 95 ℃ for 15 s, 60 ℃ for 15 s, and 72 ℃ for 10 s, for 28 cycles	WT: 250 bp<br>*KRAS*^{G12D}: 100 bp
Trp53$^{flox/flox}$	FW: GGTTAAACCCAGCTTGACCA<br>RV: GGAGGCAGAGACAGTTGGAG	95 ℃ for 30 s, 65 ℃ for 15 s (-0.5 ℃ per cycle decrease), 68 ℃ for 10 s, for 10 cycles (touch down), 95 ℃ for 15 s, 60 ℃ for 15 s, and 72 ℃ for 10 s, for 28 cycles	WT: 270 bp<br>*Trp53*^{flox}: 390 bp
Usp13$^{LSL/LSL}$	WT_FW: CACTTGCTCTCCCAAAGTCGCTC<br>Mutant_FW: AGATGTACTGCCAAGTAGGAAAGTC<br>Common_RV: ATACTCCGAGGCGGATCACAA	95 ℃ for 30 s, 60 ℃ for 35 s, and 72 ℃ for 35 s, for 30 cycles.	WT: 616 bp<br>*USP13*^{LSL}: 453 bp
