# Supplementary material for: USP13 drives lung squamous cell carcinoma by switching lung club cell lineage plasticity
Source: Mol Cancer. 2023 Dec 13;22:204. doi: 10.1186/s12943-023-01892-x (PMC10717271; doi:10.1186/s12943-023-01892-x)
Supplement: Supplementary file 5 — Additional file 5: Fig. S1. (A) Genomic alterations of USP family members in lung squamous cell carcinoma determined by cBioPortal analysis of TCGA databases (n=469). (B) Illustration of samples with 3q26 amplification in 469 LUSC patients. The 3q distal regions have been magnified to show the position of USP13, SOX2, and TP63 genes. (C) Correlation between copy number variation of USP13 and mRNA expression of squamous markers according to KRAS mutation in LUAD. Squamous markers include keratin 16 (KRT16), keratin 17 (KRT17), uroplakin 1B (UPK1B), and arachidonate lipoxygenase 3 (ALOXE3). X-axis is copy number variation of USP13 and the y-axis is log2(x+1) transformed RSEM normalized count. Error bars indicate mean ± SEM. Two-tailed unpaired t-tests, *p < 0.05, **p < 0.01. Fig. S2. (A) Schematic of KrasLSL-G12D/+; Trp53flox/flox (KP) and KrasLSL-G12D/+; Trp53flox/flox; Usp13LSL/LSL (KPU) alleles. (B) KP tumors were characterized by glandular formation (representative LUAD feature). The bottom is a high magnification of the top. Scale bar = 100 μm. (C) Different histological features in KPU tumors. (a) KPU tumors showed squamous characters such as keratin material (asterisk) and intercellular bridge (arrow). A small portion of the tumor exhibited small cell lung carcinoma (SCLC) (b) or carcinosarcoma-like histology (c).(a’), (b’) and (c’) are higher magnifications of the boxed area in (a), (b), and (c), respectively. Scale bar = 100 μm (D) H&E staining of KPU mouse lung from Figure 2C (left). Black and red lines indicate LUAD and LUSC lesions, respectively. Scale bar = 5 mm. Representative images for H&E, USP13, NKX2-1, SPC, and SOX2 IHC stains of the LUAD component in KPU tumors (right). Scale bar = 50 μm. (E) Schematic of KrasLSL-G12D/+ (K) and KrasLSL-G12D/+; Usp13LSL/LSL (KU) alleles. (F) Quantifying individual tumor number and area in K and KU mice at 55-58 weeks post-Ad-CMV-Cre infection (n= 3 and n= 5, respectively). Error bars indicate mean ± SEM. Two-tailed [file 12943_2023_1892_MOESM5_ESM.pdf]

Supplementary information

USP13 Drives Lung Squamous Cell Carcinoma via Switching Lung Club Cell Lineage Plasticity

This PDF file includes:

Figure S1

Figure S2

Figure S3

Figure S4

Figure S5

Figure S6

Figure S7

Figure S8

Figure S1

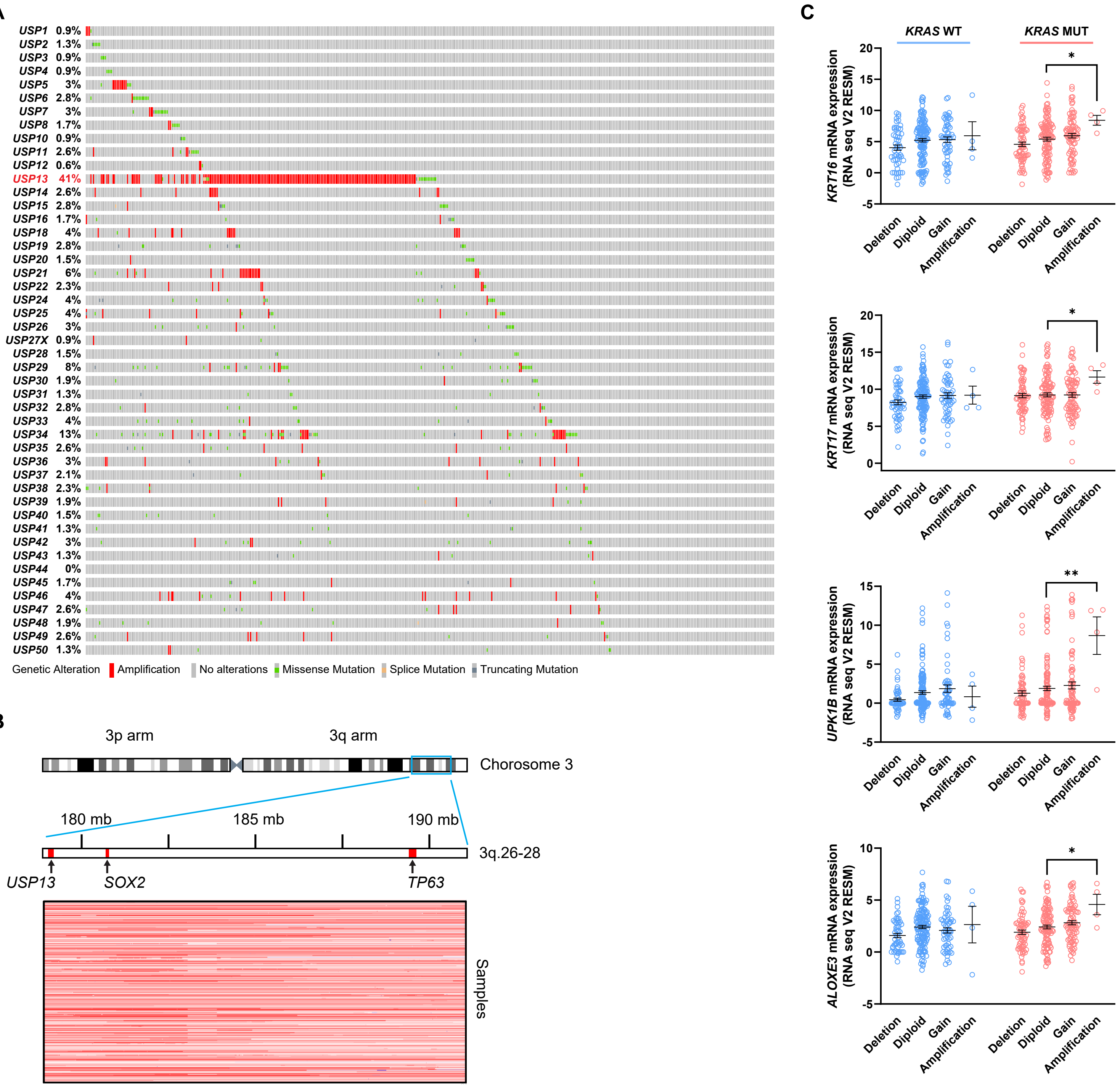
A
USP1 0.9%
USP2 1.3%
USP3 0.9%
USP4 0.9%
USP5 3%
USP6 2.8%
USP7 3%
USP8 1.7%
USP10 0.9%
USP11 2.6%
USP12 0.6%
USP13 41%
USP14 2.6%
USP15 2.8%
USP16 1.7%
USP18 4%
USP19 2.8%
USP20 1.5%
USP21 6%
USP22 2.3%
USP24 4%
USP25 4%
USP26 3%
USP27X 0.9%
USP28 1.5%
USP29 8%
USP30 1.9%
USP31 1.3%
USP32 2.8%
USP33 4%
USP34 13%
USP35 2.6%
USP36 3%
USP37 2.1%
USP38 2.3%
USP39 1.9%
USP40 1.5%
USP41 1.3%
USP42 3%
USP43 1.3%
USP44 0%
USP45 1.7%
USP46 4%
USP47 2.6%
USP48 1.9%
USP49 2.6%
USP50 1.3%
Genetic Alteration
Amplification
No alterations
Missense Mutation
Splice Mutation
Truncating Mutation
B
3p arm
3q arm
Chorosome 3
180 mb
185 mb
190 mb
3q.26-28
USP13
SOX2
TP63
Samples
C
KRAS WT
KRAS MUT
KRT16 mRNA expression (RNA seq V2 RESM)
KRT17 mRNA expression (RNA seq V2 RESM)
UPK1B mRNA expression (RNA seq V2 RESM)
ALOXE3 mRNA expression (RNA seq V2 RESM)
Deletion
Diploid
Gain
Amplification
*
**

Figure S1, related to Figure 1 (A) Genomic alterations of USP family members in lung squamous cell carcinoma determined by cBioPortal analysis of TCGA databases (n=469). (B) Illustration of samples with 3q26 amplification in 469 LUSC patients. The 3q distal regions have been magnified to show the position of *USP13*, *SOX2,* and *TP63* genes. (C) Correlation between copy number variation of *USP13* and mRNA expression of squamous markers according to *KRAS* mutation in LUAD. Squamous markers include keratin 16 (*KRT16*), keratin 17 (*KRT17*), uroplakin 1B (*UPK1B*), and arachidonate lipoxygenase 3 (*ALOXE3*). X-axis is copy number variation of *USP13* and the y-axis is log2(x+1) transformed RSEM normalized count. Error bars indicate mean ± SEM. Two-tailed unpaired t-tests, $*p < 0.05$, $**p < 0.01$.

Figure S2

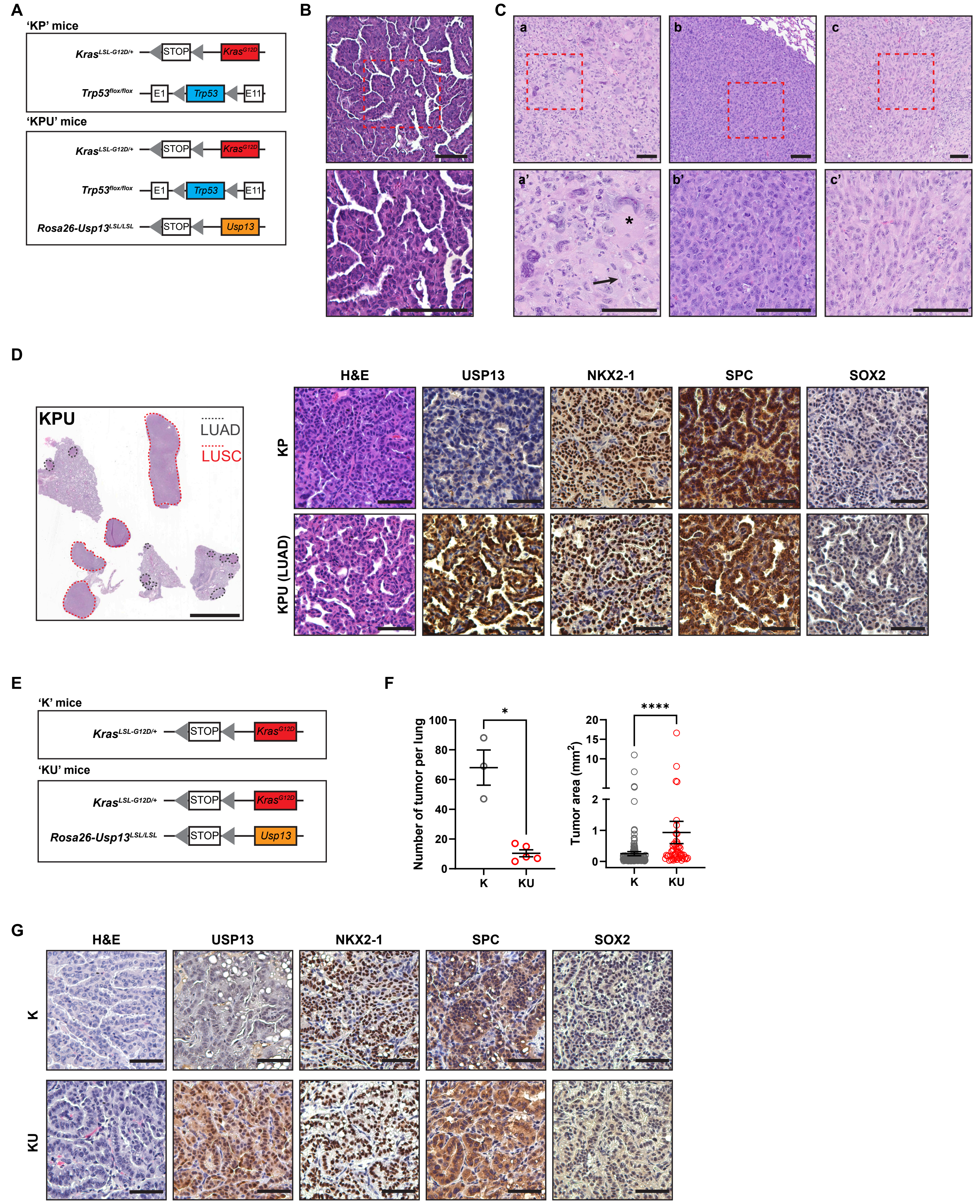
A
'KP' mice
Kras LSL-G12D/+
STOP
Kras G12D
Trp53 flox/flox
E1
Trp53
E11
'KPU' mice
Kras LSL-G12D/+
STOP
Kras G12D
Trp53 flox/flox
E1
Trp53
E11
Rosa26-Usp13 LSL/LSL
STOP
Usp13
B
C
a
b
c
a'
b'
c'
D
KPU
LUAD
LUSC
H&E
USP13
NKX2-1
SPC
SOX2
KP
KPU (LUAD)
E
'K' mice
Kras LSL-G12D/+
STOP
Kras G12D
'KU' mice
Kras LSL-G12D/+
STOP
Kras G12D
Rosa26-Usp13 LSL/LSL
STOP
Usp13
F
Number of tumor per lung
100
80
60
40
20
0
*
K
KU
Tumor area (mm2)
20
15
10
2
1
0
****
K
KU
G
H&E
USP13
NKX2-1
SPC
SOX2
K
KU

Figure S2, related to Figure 2 (A) Schematic of *Kras*$^{LSL\text{-}G12D/+}$; *Trp53*$^{flox/flox}$ (KP) and *Kras*$^{LSL\text{-}G12D/+}$; *Trp53*$^{flox/flox}$; *Usp13*$^{LSL/LSL}$ (KPU) alleles. (B) KP tumors were characterized by glandular formation (representative LUAD feature). The bottom is a high magnification of the top. Scale bar = 100 μm. (C) Different histological features in KPU tumors. (a) KPU tumors showed squamous characters such as keratin material (asterisk) and intercellular bridge (arrow). A small portion of the tumor exhibited small cell lung carcinoma (SCLC) (b) or carcinosarcoma-like histology (c). (a'), (b') and (c') are higher magnifications of the boxed area in (a), (b), and (c), respectively. Scale bar = 100 μm (D) H&E staining of KPU mouse lung from Figure 2C (left). Black and red lines indicate LUAD and LUSC lesions, respectively. Scale bar = 5 mm. Representative images for H&E, USP13, NKX2-1, SP-C, and SOX2 IHC stains of the LUAD component in KPU tumors (right). Scale bar = 50 μm. (E) Schematic of *Kras*$^{LSL\text{-}G12D/+}$ (K) and *Kras*$^{LSL\text{-}G12D/+}$; *Usp13*$^{LSL/LSL}$ (KU) alleles. (F) Quantifying individual tumor number and area in K and KU mice at 55-58 weeks post-Ad-CMV-Cre infection (n=3 and n=5, respectively). Error bars indicate mean ± SEM. Two-tailed unpaired t-tests, *p < 0.05, **p < 0.01, ***p < 0.01. (G) Representative images for H&E, USP13, NKX2-1, SP-C, SOX2 IHC stains of K and KU tumors. Scale bar = 50 μm.

Figure S3

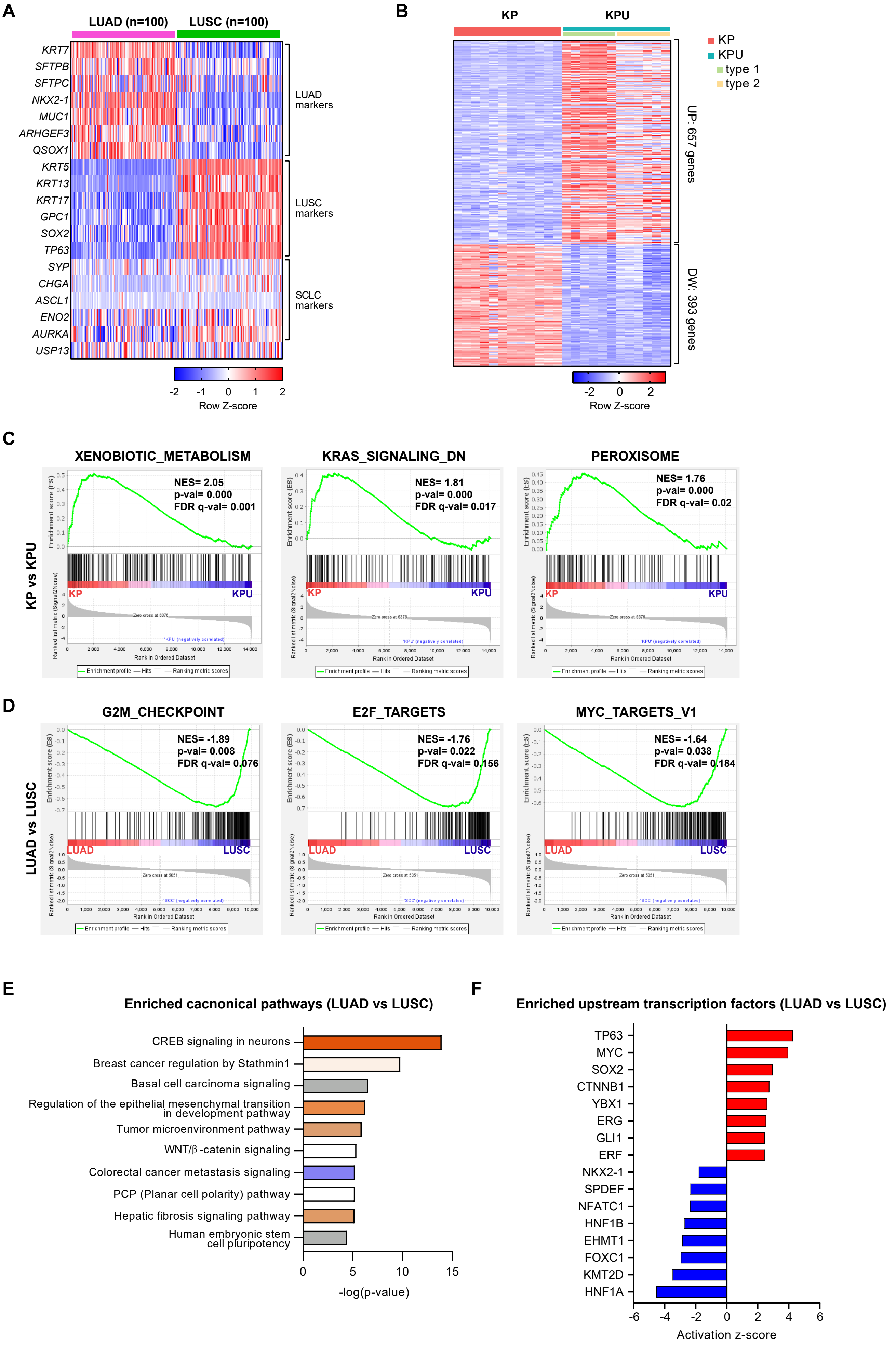
A
LUAD (n=100)
LUSC (n=100)
KRT7
SFTPB
SFTPC
NKX2-1
MUC1
ARHGEF3
QSOX1
KRT5
KRT13
KRT17
GPC1
SOX2
TP63
SYP
CHGA
ASCL1
ENO2
AURKA
USP13
LUAD markers
LUSC markers
SCLC markers
-2 -1 0 1 2
Row Z-score
B
KP
KPU
KP
KPU
type 1
type 2
UP: 657 genes
DW: 393 genes
-2 0 2
Row Z-score
C
XENOBIOTIC_METABOLISM
NES= 2.05
p-val= 0.000
FDR q-val= 0.001
KRAS_SIGNALING_DN
NES= 1.81
p-val= 0.000
FDR q-val= 0.017
PEROXISOME
NES= 1.76
p-val= 0.000
FDR q-val= 0.02
KP vs KPU
KP
KPU
Enrichment score (ES)
Ranked list metric (Signal2Noise)
Zero cross at 6376
Rank in Ordered Dataset
Enrichment profile
Hits
Ranking metric scores
D
G2M_CHECKPOINT
NES= -1.89
p-val= 0.008
FDR q-val= 0.076
E2F_TARGETS
NES= -1.76
p-val= 0.022
FDR q-val= 0.156
MYC_TARGETS_V1
NES= -1.64
p-val= 0.038
FDR q-val= 0.184
LUAD vs LUSC
LUAD
LUSC
Zero cross at 5051
Rank in Ordered Dataset
Enrichment profile
Hits
Ranking metric scores
E
Enriched cacnonical pathways (LUAD vs LUSC)
CREB signaling in neurons
Breast cancer regulation by Stathmin1
Basal cell carcinoma signaling
Regulation of the epithelial mesenchymal transition in development pathway
Tumor microenvironment pathway
WNT/β-catenin signaling
Colorectal cancer metastasis signaling
PCP (Planar cell polarity) pathway
Hepatic fibrosis signaling pathway
Human embryonic stem cell pluripotency
0 5 10 15
-log(p-value)
F
Enriched upstream transcription factors (LUAD vs LUSC)
TP63
MYC
SOX2
CTNNB1
YBX1
ERG
GLI1
ERF
NKX2-1
SPDEF
NFATC1
HNF1B
EHMT1
FOXC1
KMT2D
HNF1A
-6 -4 -2 0 2 4 6
Activation z-score

Figure S3, related to Figure 3 (A) Heatmap showing the expression of marker genes for LUAD, LUSC, and SCLC in TCGA LUAD and LUSC samples. (B) Heatmap visualization of differentially expressed genes (DEGs) ($|log_2FC| > 3.5$, $p < 0.01$, and $q < 0.01$) between KP and KPU tumor samples. (C) Enrichment plots for hallmark xenobiotic metabolism, KRAS signaling down, and peroxisome for KP and KPU tumors. (D) GSEA analysis for hallmark G2M checkpoint, E2F targets, and MYC targets version 1 for LUAD and LUSC. (E) IPA analysis shows the top ten canonical pathways enriched in LUSC compared with LUAD. Each bar's color indicates predicted pathway activation or inhibition; Orange, positive z-score (activation); white, zero z-score; blue, negative z-score (inhibition); gray, no pattern. (F) Upstream regulator analysis exhibits transcription factors' predicted activation status in human LUSC. A positive z-score indicates activation and a negative z-score indicates inhibition.

Figure S4

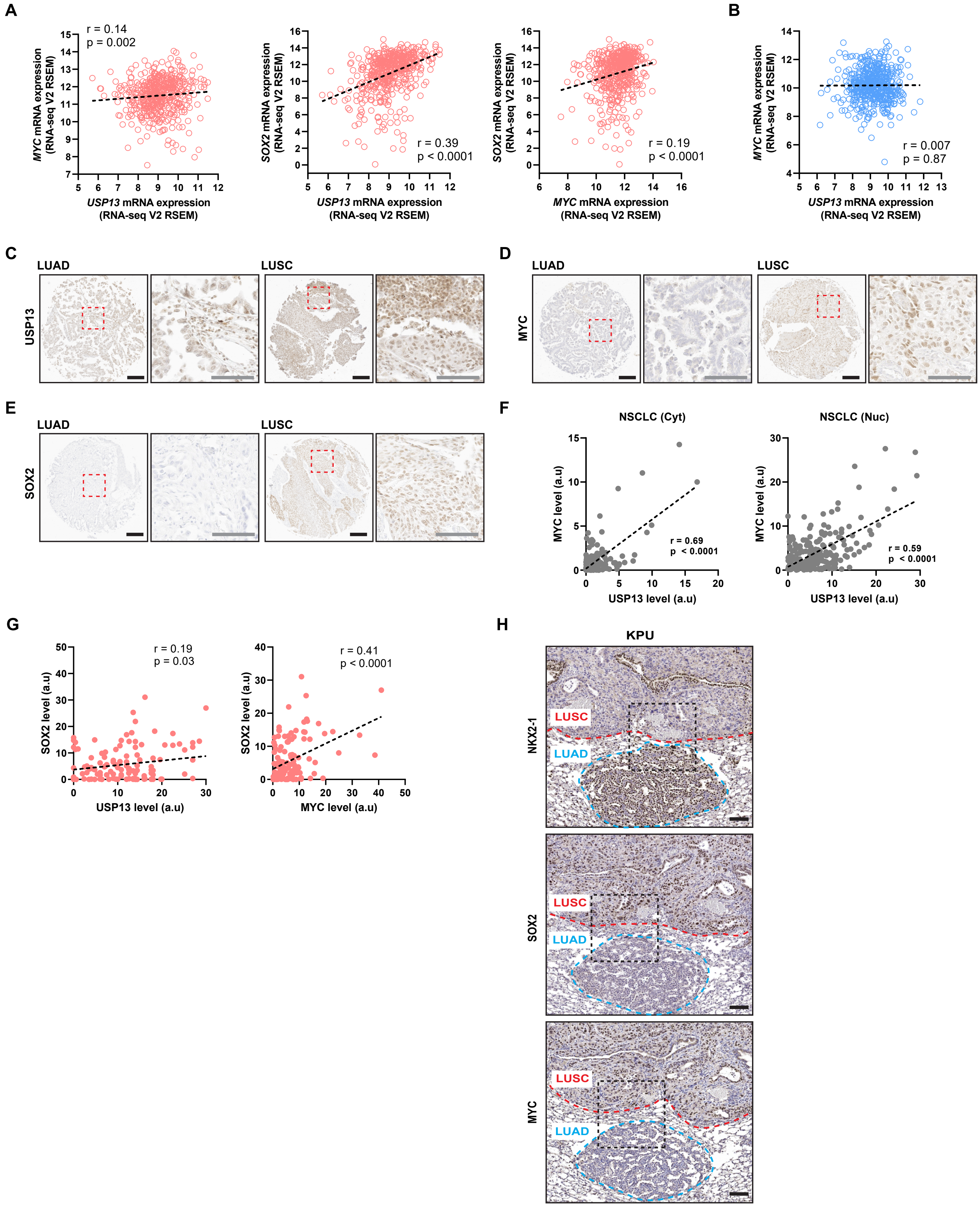
A
r = 0.14
p = 0.002
MYC mRNA expression (RNA-seq V2 RSEM)
USP13 mRNA expression (RNA-seq V2 RSEM)
r = 0.39
p < 0.0001
SOX2 mRNA expression (RNA-seq V2 RSEM)
USP13 mRNA expression (RNA-seq V2 RSEM)
r = 0.19
p < 0.0001
SOX2 mRNA expression (RNA-seq V2 RSEM)
MYC mRNA expression (RNA-seq V2 RSEM)
B
r = 0.007
p = 0.87
MYC mRNA expression (RNA-seq V2 RSEM)
USP13 mRNA expression (RNA-seq V2 RSEM)
C
LUAD
LUSC
USP13
D
LUAD
LUSC
MYC
E
LUAD
LUSC
SOX2
F
NSCLC (Cyt)
NSCLC (Nuc)
MYC level (a.u)
USP13 level (a.u)
r = 0.69
p < 0.0001
r = 0.59
p < 0.0001
G
r = 0.19
p = 0.03
SOX2 level (a.u)
USP13 level (a.u)
r = 0.41
p < 0.0001
SOX2 level (a.u)
MYC level (a.u)
H
KPU
NKX2-1
SOX2
MYC
LUSC
LUAD

Figure S4, related to Figure 4 (A) Correlation between *USP13* and *MYC* mRNA expression (left), *USP13* and *SOX2* mRNA expression (center), and *MYC* and *SOX2* mRNA expression (right) in LUSC. Data was obtained from cBioportal (TCGA, PanCancer Atlas) (n=469). mRNA Expression is log2(x+1) transformed RSEM normalized count. (B) Correlation between *USP13* and *MYC* mRNA expression in LUAD. Data was obtained from cBioportal (TCGA, PanCancer Atlas) (n=507). (C-E) Representative IHC-staining images of USP13 (C), MYC (D), and SOX2 (E) in LUAD (left) and LUSC (right). The red boxed areas on the left images were magnified and shown on the right. Black scale bar = 100 μm, Greyscale bar = 50 μm. (F) Correlation between USP13 and MYC in the cytoplasm (left) and nucleus (right) of NSCLC patient samples. (G) Correlation between USP13 and SOX2 expression (left) and MYC and SOX2 expression (right) in LUSC. Data was obtained from Figure 4B (n=130). IHC scores are analyzed using a two-tailed Pearson correlation coefficient. (H) Lower magnification images of Figure 4F. Scale bar = 200 μm.

Figure S5

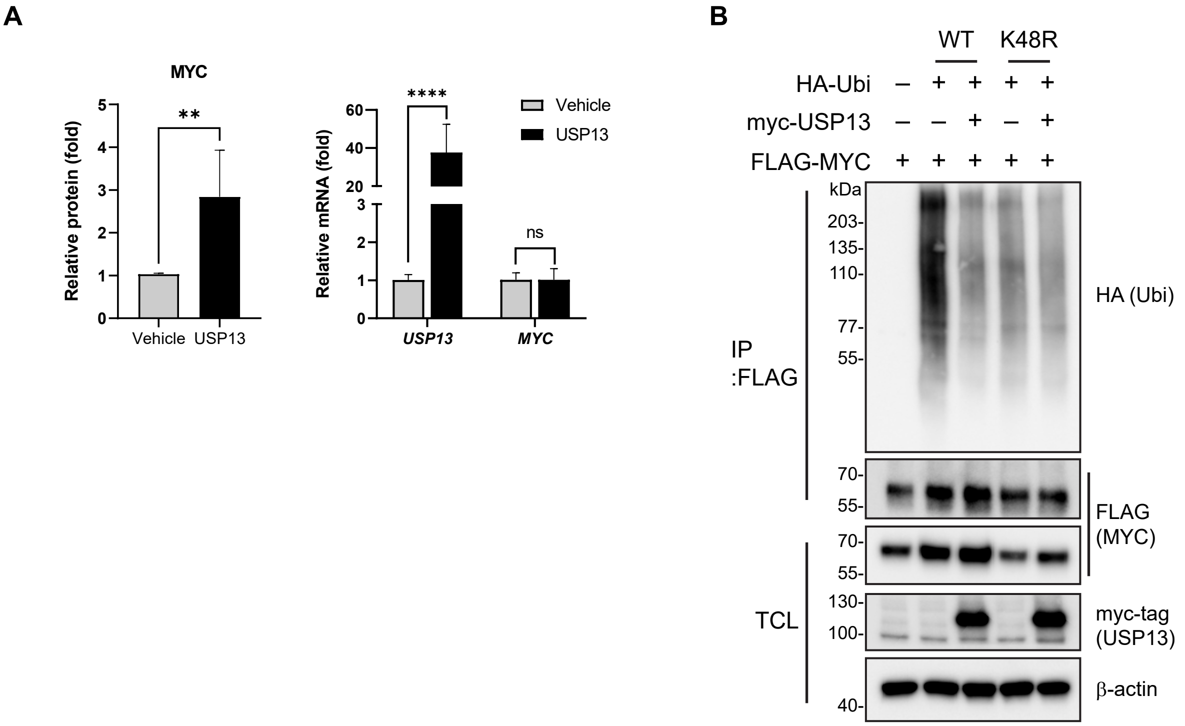
A
MYC
Relative protein (fold)
**
Vehicle
USP13
Relative mRNA (fold)
****
ns
USP13
MYC
Vehicle
USP13
B
WT
K48R
HA-Ubi
myc-USP13
FLAG-MYC
kDa
203-
135-
110-
77-
55-
70-
55-
130-
100-
40-
IP
:FLAG
TCL
HA (Ubi)
FLAG
(MYC)
myc-tag
(USP13)
β-actin

Figure S5, related to Figure 6 (A) Quantification of MYC expression levels relative to the vehicle is shown (left). qPCR of *USP13* and *MYC* mRNA expression in HEK293T cells with or without USP13 overexpression. mRNA level was normalized to β-actin. Data are shown as means ± SD. ns, not significant, $**p < 0.01$, and $****p < 0.0001$ (unpaired two-tailed t-test). (B) USP13-mediated deubiquitylation acts upon the Lys48 (K48) ubiquitination of MYC. 293T cells were transfected with FLAG-MYC, HA-Ub (WT or K48R), and Myc-tagged USP13.

Figure S6

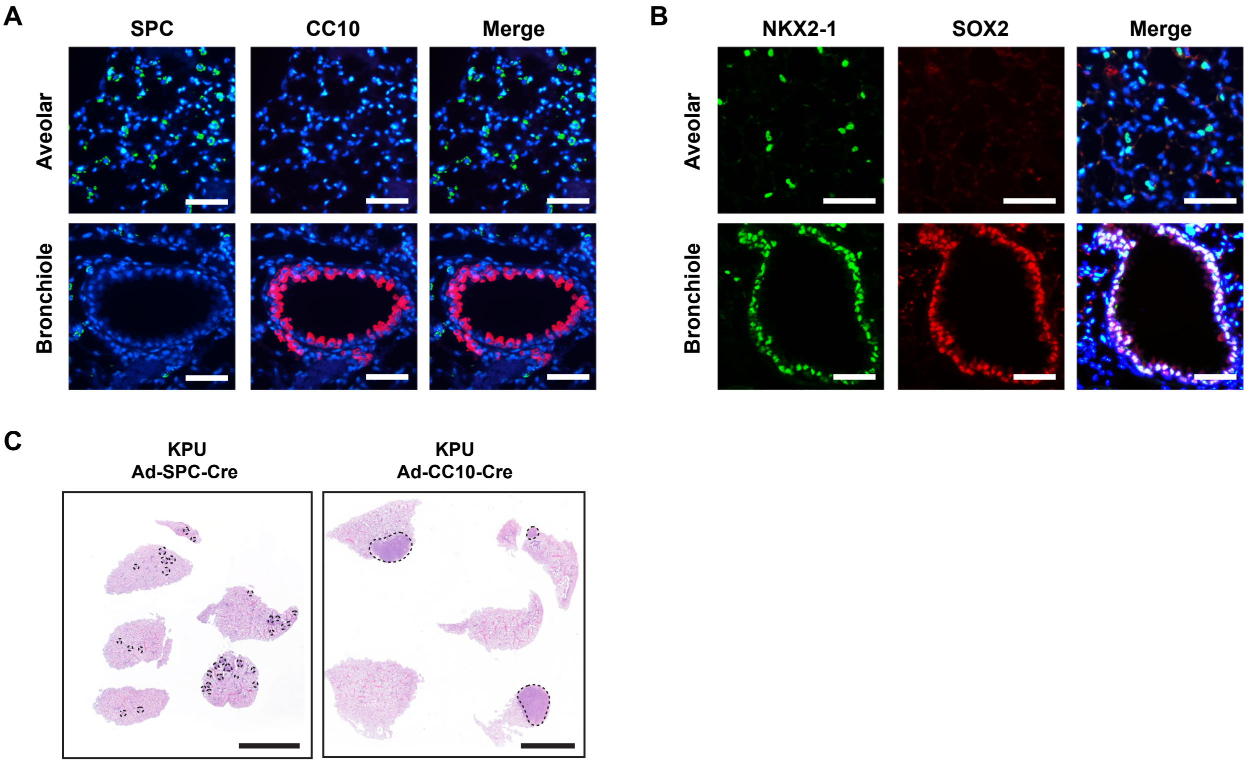
A
SPC
CC10
Merge
Aveolar
Bronchiole
B
NKX2-1
SOX2
Merge
Aveolar
Bronchiole
C
KPU
Ad-SPC-Cre
KPU
Ad-CC10-Cre

Figure S6, related to Figure 6 (A) Section of wild-type mouse lung stained with anti-SPC (green) and anti-CC10 (red). Scale bar = 50 μm. (B) Section of wild-type mouse lung stained with anti-NKX2-1 (green) and anti-SOX2 (red). Scale bar = 50 μm. (C) Representative hematoxylin and eosin (H&E) staining of KPU lung infected by Ad-SPC-Cre or Ad-CC10-Cre virus. Dotted lines indicate tumor nodules. Scale bar = 5 mm.

Figure S7

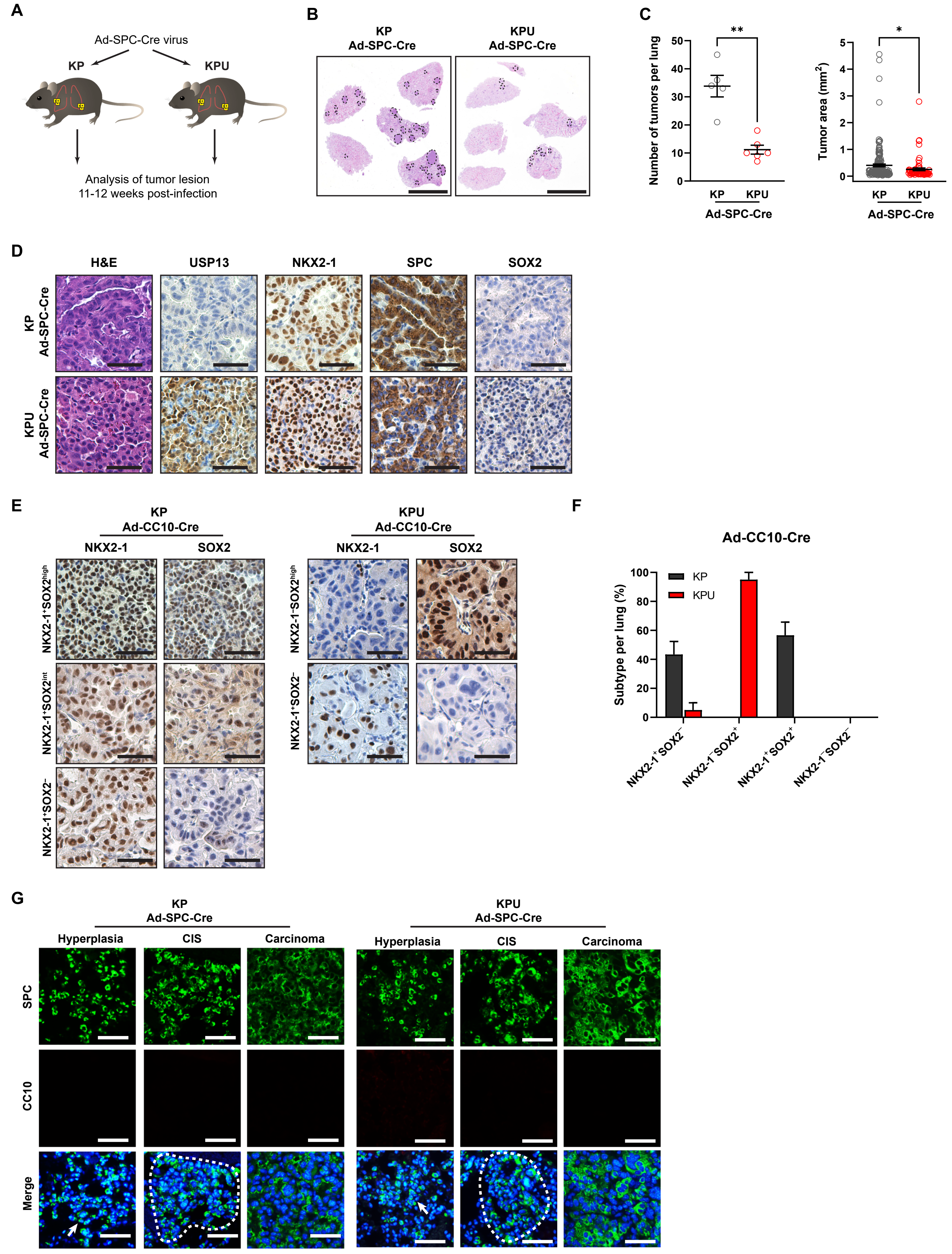
A
Ad-SPC-Cre virus
KP
KPU
Analysis of tumor lesion
11-12 weeks post-infection
B
KP
Ad-SPC-Cre
KPU
Ad-SPC-Cre
C
Number of tumors per lung
**
KP
KPU
Ad-SPC-Cre
Tumor area (mm2)
*
KP
KPU
Ad-SPC-Cre
D
H&E
USP13
NKX2-1
SPC
SOX2
KP
Ad-SPC-Cre
KPU
Ad-SPC-Cre
E
KP
Ad-CC10-Cre
NKX2-1
SOX2
KPU
Ad-CC10-Cre
NKX2-1
SOX2
NKX2-1+SOX2high
NKX2-1+SOX2int
NKX2-1+SOX2−
NKX2-1−SOX2high
NKX2-1+SOX2−
F
Ad-CC10-Cre
KP
KPU
Subtype per lung (%)
NKX2-1+SOX2−
NKX2-1−SOX2+
NKX2-1+SOX2+
NKX2-1−SOX2−
G
KP
Ad-SPC-Cre
Hyperplasia
CIS
Carcinoma
KPU
Ad-SPC-Cre
Hyperplasia
CIS
Carcinoma
SPC
CC10
Merge

Figure S7, related to Figure 7 (A) Experiment design: KP or KPU mice received a single intratracheal injection of cell-type-restricted Ad-SPC-Cre virus. The experiment was terminated 11-12 weeks post-viral infection. (B) Representative hematoxylin and eosin (H&E) staining of KP and KPU lung at 12 weeks post-Ad-SPC-Cre infection. Dotted line indicates a tumor nodule. Scale bar = 5 mm. (C) Quantification of individual tumor number (left) and area (right) in KP and KPU mice at 11-12 weeks post-Ad-SPC-Cre virus infection (n=5 mice/group). (D) Representative images for H&E, USP13, NKX2-1, SP-C, and SOX2 IHC stains of the KP and KPU tumors. Scale bar = 50 μm. (E) Representative NKX2-1 and SOX2 staining images from lung tumors in Ad-CC10-Cre infected KP (left) and KPU (right) mice. Scale bar = 50 μm. (F) Quantification of lesions with NKX2-1 and SOX2 expression patterns from (E). (G) SPC and CC10 expression during cancer progression in Ad-SPC-Cre infected KP and KPU lungs. Hyperplasia (arrow) and carcinoma *in situ* (CIS) (dotted line) are indicated. Scale bar = 50 μm. In (C) and (F), error bars indicate mean ± SEM. Two-tailed unpaired t-tests, $^*p < 0.05$, $^{**}p < 0.01$.

Figure S8

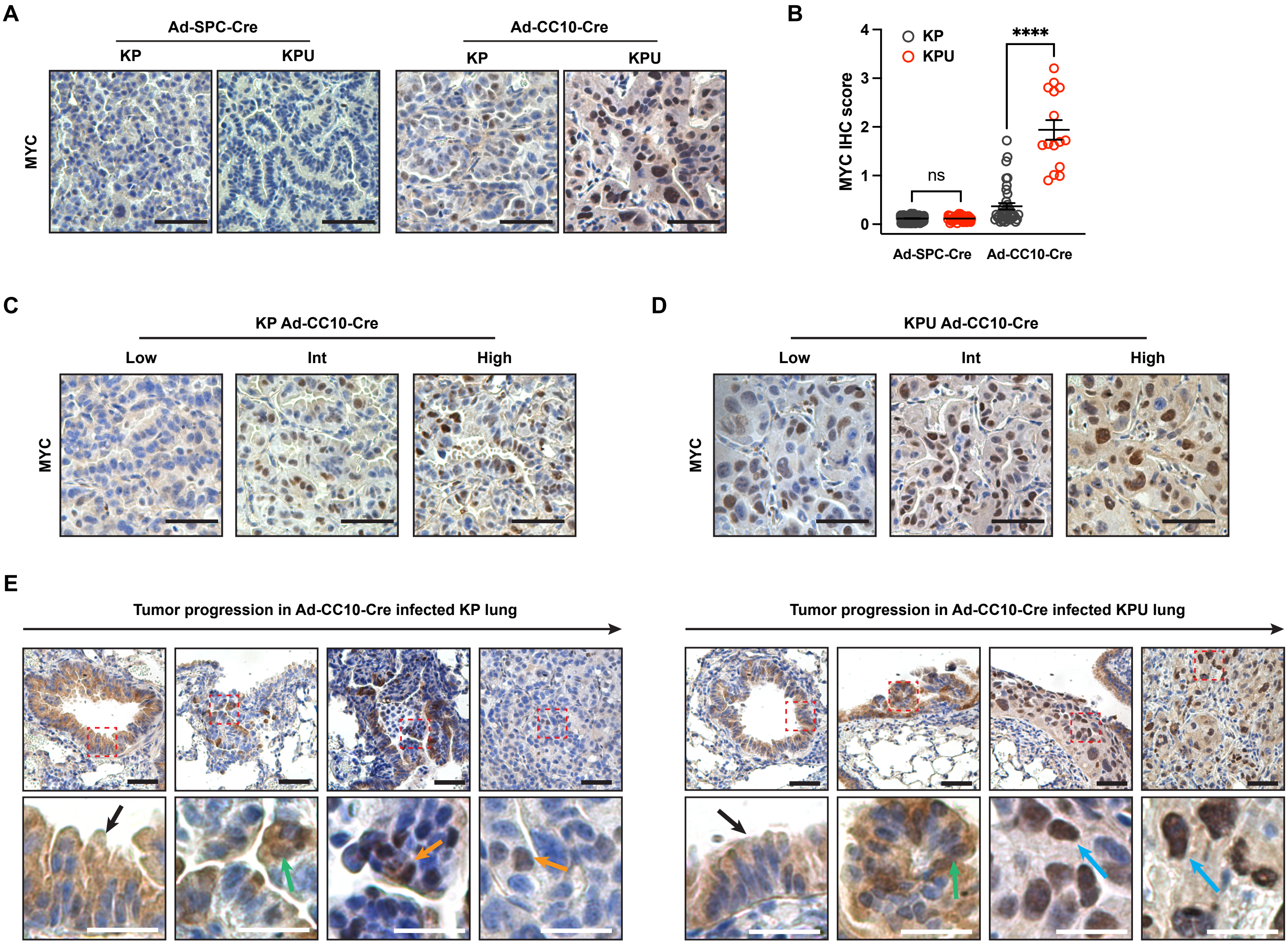
A
Ad-SPC-Cre
KP
KPU
Ad-CC10-Cre
KP
KPU
MYC
B
KP
KPU
****
ns
MYC IHC score
0
1
2
3
4
Ad-SPC-Cre
Ad-CC10-Cre
C
KP Ad-CC10-Cre
Low
Int
High
MYC
D
KPU Ad-CC10-Cre
Low
Int
High
MYC
E
Tumor progression in Ad-CC10-Cre infected KP lung
Tumor progression in Ad-CC10-Cre infected KPU lung

Figure S8, related to Figure 8 (A) Representative images for IHC staining of MYC in the KP and KPU tumors post-Ad-SPC-Cre (left) or Ad-CC10-Cre (right) infection. Scale bar = 50 µm (B) IHC quantification for MYC in tumors. Error bars indicate mean ± SEM. Two-tailed unpaired t-tests, ns = not significant, ****$p < 0.0001$. (C and D) Representative images of KP (C) and KPU (D) tumors with low, intermediate, and high MYC expression. Scale bar = 50 µm. (E) Cellular localization of MYC during tumor progression in KP (left) and KPU (right) lungs following Ad-CC10-Cre infection. The bottom figures are higher magnifications of the boxed area in the top figures. Arrows indicate the cellular location of MYC protein; Black, cytoplasm; Green, whole cell; Orange, nucleus; Cyan, whole cell with nuclear enrichment. Black scale bar = 50 µm, white scale bar = 25 µm.
